# Takotsubo syndrome and COVID‐19: A systematic review

**DOI:** 10.1002/hsr2.972

**Published:** 2022-12-02

**Authors:** Hoomaan Ghasemi, Sina Kazemian, Seyed Aria Nejadghaderi, Mahan Shafie

**Affiliations:** ^1^ School of Medicine Tehran University of Medical Sciences Tehran Iran; ^2^ Students' Scientific Research Center (SSRC) Tehran University of Medical Sciences Tehran Iran; ^3^ Cardiac Primary Prevention Research Center (CPPRC), Tehran Heart Center Tehran University of Medical Sciences Tehran Iran; ^4^ Research Center for Integrative Medicine in Aging, Aging Research Institute Tabriz University of Medical Sciences Tabriz Iran; ^5^ Systematic Review and Meta‐Analysis Expert Group (SRMEG) Universal Scientific Education and Research Network (USERN) Tehran Iran

**Keywords:** COVID‐19, SARS‐CoV‐2, stress cardiomyopathy, systematic review, takotsubo cardiomyopathy, takotsubo syndrome

## Abstract

**Background and Aims:**

Takotsubo syndrome (TTS), also known as stress cardiomyopathy, is characterized by acute and transient left ventricular dysfunction and has increased during the COVID‐19 pandemic. Herein, we aim to review studies on TTS that were associated with COVID‐19 infection, vaccine, and other COVID‐19‐related etiologies including psychosocial stressors.

**Methods:**

We systematically searched PubMed, EMBASE, and Scopus up to May 12, 2022. We included case reports, case series, and original articles that reported at least one TTS case associated with COVID‐19, or TTS cases after receiving COVID‐19 vaccines, or TTS cases secondary to psychological stress due to the COVID‐19 pandemic. The quality assessment was conducted using the Joanna Briggs Institute checklist.

**Results:**

Sixty‐seven articles including 102 cases were included. Hypertension was the most frequently accompanying comorbidity (*N* = 67 [65.6%]) and the mean left ventricular ejection fraction was 36.5%. Among COVID‐19 patients, the in‐hospital mortality rate was 33.3%. On the other hand, only one COVID‐19‐negative individual expired (2.3%). The most common presenting clinical symptom was dyspnea in 42 (73.6%) patients. the mean time interval from the first symptom to admission was 7.2 days. The most common chest imaging finding was ground‐glass opacity which was reported in 14 (31.1%) participants. The most common abnormalities were T‐wave inversion in 35 (43.2%) and ST‐segment elevation in 30 (37%). Brain natriuretic peptide and troponin were elevated in 94.7% and 95.9% of participants, respectively.

**Conclusion:**

The TTS in patients with COVID‐19 is almost rare, whereas it could lead to a great mortality and morbidity. An individual with COVID‐19, especially an elderly woman, presented with dyspnea in addition to a rise in brain natriuretic peptide and troponin should be evaluated for TTS.

## 1 INTRODUCTION

Takotsubo syndrome (TTS) also known as stress cardiomyopathy is characterized by acute and transient left ventricular dysfunction without coronary obstruction.^1^ It is reported to be often triggered by physical or emotional triggers.^1^ The clinical presentation of TTS closely imitates that of acute coronary syndrome, which most patients present with chest pain, show ST‐segment elevation on electrocardiogram (ECG), and mild increase in serum troponin levels.^2^ Due to a similar clinical picture of TTS and acute coronary syndrome, distinguishing the two conditions still remains crucial; therefore, ischemic cardiomyopathies have to be excluded before TTS diagnosis.^2^ Although TTS is often reversible, it can lead to acute heart failure, left ventricle outflow tract obstruction, cardiogenic shock, thrombosis formation, and arrhythmias.^3^

Recently, studies have reported an increase in TTS incidence during the COVID‐19 pandemic.^4^ A retrospective cohort study indicated that acute coronary syndrome resulting from stress cardiomyopathy was found to be higher during the pandemic period (7.8%) compared to similar periods before the pandemic (1.5%–1.8%).^5^ These observations suggested viral mechanism associated with COVID‐19 causing TTS, as well as an increase in TTS in the pandemic due to the associated psychological, social, and economic stress derived from imposed restrictive measures. Therefore, the severe acute respiratory syndrome coronavirus disease 2 (SARS‐CoV‐2) infection potentially induces physical and psychosocial stress in patients, which may lead to an increase in the risk of TTS development.^6^ Although the underlying pathophysiology of COVID‐19‐induced TTS still remains unclear, some mechanisms have been proposed in this regard. Direct viral myocardial injury, downregulation of angiotensin‐converting enzyme 2 receptors in myocardium, cytokine storm, surge in catecholamines, and vascular inflammation are proposed to be associated with cardiac injury.^7^, ^8^, ^9^, ^10^ Moreover, studies also reported TTS and other cardiomyopathies in non‐COVID‐19 patients after receiving the mRNA‐based and other vaccines despite the low reported rate of cardiovascular complications.^11^

A previous systematic review evaluated the effects of COVID‐19 in development of TTS using case reports in 2020.^12^ The study did not evaluate the effects of COVID‐19 vaccination in the development of TTS and its results need to be updated. Hence, in this systematic review, we aimed to review studies on TTS that were associated with COVID‐19 infection, vaccine, and other COVID‐19‐related etiologies including psychosocial stressors. The findings could be helpful for clinicians and health authorities for prevention and management of TTS in the COVID‐19 pandemic.

## 2 METHODS

The present systematic review was prepared based on Preferred Reporting Items for Systematic reviews and Meta‐Analyses guidelines.^13^ The study protocol was approved by PROSPERO with the registration code CRD42021282245 (www.crd.york.ac.uk/PROSPERO/). Since ethical approval and the Institutional Review Board (IRB) were reported for each of the included studies, no additional ethical or IRB approvals were required for this systematic review.

### 2.1 Search strategy

We searched the main medical databases, including PubMed, EMBASE and Scopus, up to May 12, 2022. We used following search terms and combinations: (“takotsubo cardiomyopathy” OR “takotsubo syndrome” OR “stress cardiomyopathy” OR “broken heart syndrome” OR “apical ballooning syndrome”) AND (“COVID‐19” OR “2019‐nCoV disease” OR “coronavirus disease‐19” OR “SARS Coronavirus 2” OR “SARS‐CoV‐2” OR “Wuhan Coronavirus” OR “COVID‐19 Vaccines” OR “SARS‐CoV‐2 Vaccine” OR “Coronavirus Disease 2019 Vaccine” OR “2019‐nCoV Vaccine”). The search strategy for each database is provided in Supporting Information: Table S1. Neither article language nor publication time was restricted. All of the titles and abstracts were screened independently by three reviewers to find potentially eligible studies. Discussions among all of the authors resolved disagreements regarding the inclusion of studies. The full text of those studies found to be eligible for inclusion based on the title and abstract screening were further studied and assessed for inclusion. Backward and forward citation searching was conducted to find any potential additional studies.

### 2.2 Inclusion and exclusion criteria

We included case reports and case series that reported at least one TTS case associated with COVID‐19, or TTS cases after receiving COVID‐19 vaccines, or TTS cases secondary to psychological stress due to the COVID‐19 pandemic. We also included all observational studies, including, case–control, cohort, and cross‐sectional studies, that investigate the association of TTS to COVID‐19 infection and other COVID‐19‐related stressors. We excluded opinions, book chapters, reviews, letters, and conference abstracts, as well as animal and in‐vitro studies. The diagnosis of TTS is considered in accordance with the criteria outlined by Mayo Clinic.^14^

### 2.3 Data extraction

Three authors separately extracted the following data from studies: study characteristics including author name, publication date, study design, study country, inclusion and exclusion criteria, number of participants and cases, demographic data of participants including age, gender, race and ethnicity, body mass index (BMI), history of menopause, history of neuropsychological disorders, history of cardiovascular risk factors including diabetes, hypertension, and dyslipidemia, history of myocardial infarction, any other medical history, vital signs during presentation including heart rate, blood pressure, respiratory rate, body temperature, and O_2_ saturation, examination findings, COVID‐19 test results, COVID‐19 symptoms including fever, dyspnea, chest pain, cough, or other presentations, troponin, brain natriuretic peptide (BNP), and creatinine kinase (CK) level, electrocardiogram findings, trans‐thoracic echocardiogram findings, angiography findings, type of TTS, and outcomes. The discrepancies were resolved by discussion or consultation with another author.

### 2.4 Quality assessment

The quality of all included studies was assessed using the Joanna Briggs Institute (JBI) critical appraisal checklists for case reports,^15^ case series,^16^ and cohort studies.^17^ The checklist for case reports rates the quality of studies by eight major questions which are providing the patient's demographic characteristics, medical history, current clinical condition, description of diagnostic tests, treatment, post‐intervention clinical conditions, adverse events, and mentioning of takeaway lessons.^15^ The checklist for case series include items which are providing the inclusion criteria, methods of condition measurement, validity of the diagnostic methods, consecutive inclusion of participants, completeness of participants' inclusion, reporting of the demographic characteristics, clinical information, outcomes, presenting clinic demographic information and the quality of the statistical analysis.^16^ The JBI checklist for cohort studies includes 11 items, which are similarity of the two groups, similarity in measurement of exposures, validity and reliability of measuring exposure, identifying confounders, stating strategies for dealing with confounders, lacking of the outcomes at the start of the study, validity and reliability of outcome measurement, completeness and sufficient duration of follow‐up time, using strategies to address incomplete follow‐up, and using appropriate statistical analysis.^17^ A higher quality score represents a better quality of that study in the JBI checklists.

## 3 RESULTS

**Figure 1:**
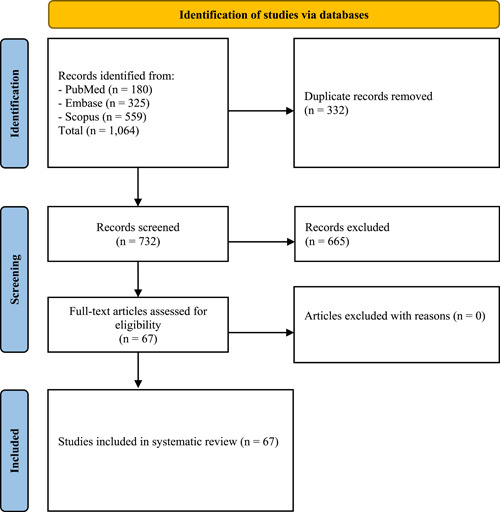
Study selection process

A total of 1064 studies were identified. After applying the eligibility criteria and title and abstract review followed by detailed evaluations, 67 articles were selected.^4^, ^5^, ^9^, ^11^, ^18^, ^19^, ^20^, ^21^, ^22^, ^23^, ^24^, ^25^, ^26^, ^27^, ^28^, ^29^, ^30^, ^31^, ^32^, ^33^, ^34^, ^35^, ^36^, ^37^, ^38^, ^39^, ^40^, ^41^, ^42^, ^43^, ^44^, ^45^, ^46^, ^47^, ^48^, ^49^, ^50^, ^51^, ^52^, ^53^, ^54^, ^55^, ^56^, ^57^, ^58^, ^59^, ^60^, ^61^, ^62^, ^63^, ^64^, ^65^, ^66^, ^67^, ^68^, ^69^, ^70^, ^71^, ^72^, ^73^, ^74^, ^75^, ^76^, ^77^, ^78^, ^79^, ^80^ Of these articles, 46 were centered around TTS in patients with COVID‐19, 13 were related to TTS in patients with emotional triggering events, and 8 were linked to TTS in patients with recent COVID‐19 vaccination (Figure 1).

All articles were written in English. Thirty (44.7%) were from the United States. The details on the study characteristic of included articles are represented in Table 1. A total of 102 patients were included in this review which comprised three groups. The first group consists of 60 patients (58.8%) with confirmed COVID‐19 diagnosis, the second group consisted of 34 cases (33.3%) with known emotional triggering events, and the third group consisted of 8 cases (7.8%) who had recently received COVID‐19 vaccination. The mean age of the reported cases was 67.2 (SD = 12.6; range 30–94) years with a female predominance of 68.6%. Among women with TTS, data on patients' age was available in 57 cases, of which 78.9% were above 60 years of age. Hypertension was the most frequently accompanying comorbidity (*N* = 67 [65.6%]), followed by dyslipidemia (*N* = 38 [37.2%]) and diabetes (*N* = 26 [25.4%]). Data on the left ventricular ejection fraction (LVEF) were reported in 60 cases. The overall mean LVEF was 36.5% (males: 37.2% and females: 36.2%) (Table 1). Among COVID‐19 patients, the in‐hospital mortality rate was 33.3%. On the other hand, only one COVID‐19‐negative individual was expired (2.3%) (Table 1).

**Table 1:** Baseline and clinical characteristics of the reported patients

Study, year	Study design	Study country	Age (years)	Gender	Past medical history	LVEF (%)	Outcome	Follow‐up echocardiography
Takotsubo after COVID‐19 articles								
Nguyen et al. (2020)^19^	Case report	Belgium	71	Female	HTN, DLP	NR	Discharged in good condition	NR
Panchal et al. (2020)^20^	Case report	USA	65	Male	DM, HTN, AF	NR	Deceased	N/A
Kariyanna et al. (2020)^21^	Case report	USA	72	Female	DM, HTN, DLP, obesity	NR	Deceased	N/A
Alizadehasl et al. (2022)^22^	Case report	Iran	57	Male	HTN, DLP	NR	Discharged in good condition	On 6 weeks follow‐up: mild biventricular residual systolic dysfunction
Fujisaki et al. (2021)^23^	Case report	USA	60	Male	DM, HTN, DLP	15%	Discharged in good condition	On 37 days follow‐up: resolution of the wall motion abnormalities and the LVEF was 55%
Demertzis et al. (2020)^24^	Case series	USA	76	Female	HTN, obstructive sleep apnea	40%	Deceased	N/A
			67	Female	Nonischemic dilated cardiomyopathy with preservedEF = 50%, HTN	N/A	Discharged in good condition	On 3 weeks follow‐up: resolution of the wall motion abnormalities with an EF of 63%
Torabi et al. (2021)^25^	Case report	USA	42	Female	Crohn's disease	20%	Deceased	N/A
Ortuno et al. (2021)^26^	Case report	France	79	Male	DM, HTN, CKD	40%	Deceased	Restoration of LVEF with decrease of apical ballooning aspect
Hegde et al. (2020)^27^	Case series	USA	71	Female	DM, HTN, DLP	15%	Deceased	N/A
			78	Male	DM, HTN, DLP, CVA, AF	53%	Discharged in good condition	NR
			70	Female	DM, HTN, DLP	45%	Discharged in good condition	NR
			78	Female	DM, HTN, DLP, CVA, AF	20%	Deceased	N/A
			88	Male	DM, HTN, DLP, CKD, CVA, AF	30%	Deceased	N/A
			58	Male	DLP	40%	Discharged in good condition	NR
			56	Male	HTN, DLP, CVA, AF, schizophrenia	45%	Deceased	N/A
Hoepler et al. (2021)^28^	Case series	Austria	67	Female	HTN, DLP, CKD	65%	Discharged in good condition	On 4 weeks follow‐up: normal left ventricular function without any regional differences and normal heart valves
			60	Female	COPD, depression	68%	Still under medical care	NR
			73	Female	Osteoporosis, chronic pain syndrome	20%	Still under medical care	On 2 weeks follow‐up: normal global systolic left ventricular function
Alshamam et al. (2021)^4^	Case report	USA	86	Female	HTN, osteoporosis, anemia	35%‐40%	Deceased	N/A
Bernardi et al. (2020)^29^	Case report	Italy	74	Male	Impaired fasting blood sugar, HTN, DLP	30%	Discharged in good condition	On 14 days follow‐up: resolution of the 2 thrombi and a complete restoration of LVEF (57%)
Sattar et al. (2020)^30^	Case report	USA	67	Female	DM, HTN	30%	Discharged in good condition	NR
Tsao et al. (2020)^31^	Case report	USA	59	Female	None	36%	Discharged in good condition	On 10 days follow‐up: resolution of the stress cardiomyopathy, with normal biventricular systolic function
Gomez et al. (2020)^32^	Case report	USA	57	Female	Crohn's disease, morbid obesity	25%–30%	Discharged in good condition	On 18 days follow‐up: resolution of left ventricular dysfunction with no appreciable regional wall abnormalities (LVEF 70%–75%)
Belli et al. (2021)^18^	Case report	Italy	53	Female	CKD	30%	Still under medical care	On following week follow‐up: improvement of left ventricular systolic function and motion abnormalities
Titi et al. (2021)^33^	Case report	Italy	83	Male	DM, HTN, DLP, COPD	NR	Deceased	N/A
Faqihi et al. (2020)^34^	Case report	Kingdom of Saudi Arabia	40	Male	None	30%	Discharged in good condition	NR
Solano‐López et al. (2020)^35^	Case report	Spain	50	Male	Asymptomatic benign mediastinal tumor since childhood	NR	Discharged in good condition	On discharge: significant improvement of left ventricular contractility
Pasqualetto et al. (2020)^36^	Case series	Italy	84	Male	DM, HTN	53%	Discharged in good condition	NR
			85	Female	HTN	30%	Deceased	N/A
			81	Male	DM, HTN	42%	Discharged in good condition	NR
Koh et al. (2021)^37^	Case report	Singapore	34	Male	None	30%	Discharged in good condition	NR
Dave et al. (2020)^38^	Case report	USA	59	Female	HTN, COPD	26%	Deceased	N/A
Van Osch et al. (2020)^39^	Case report	UK	72	Female	AF	30%	Discharged in good condition	On 3 months follow‐up: normal contractility of the apical myocardial segments, with normalization of the left ventricular systolic function (LVEF: 55%)
Bhattacharyya et al. (2020)^40^	Case report	India	32	Female	None	38%	Discharged in good condition	On 13 days follow‐up: normalization of the LV regional wall motion abnormalities (LVEF: 51%)
Taza et al. (2020)^41^	Case report	USA	52	Male	DM, HTN, schizophrenia	45%	Discharged in good condition	NR
Bottiroli et al. (2020)^42^	Case report	Italy	76	Female	None	25%	Discharged in good condition	On 62 days follow‐up: improvement of the wall motion of apical segments and complete recovery of LVEF up to normal values
Roca et al. (2020)^43^	Case report	Italy	87	Female	Breast cancer	48%	Discharged in good condition	NR
Oyarzabal et al. (2020)^44^	Case report	Spain	82	Male	DM, HTN, DLP, CKD	NR	Discharged in good condition	NR
Minhas et al. (2020)^45^	Case report	USA	58	Female	DM, HTN, DLP	20%	Discharged in good condition	On 6 days follow‐up: improvement noted in overall wall motion and LVEF 55%
Kong et al. (2021)^46^	Case series	USA	88	Male	Prostate cancer under chemotherapy, dementia	Severe LV systolic dysfunction	Deceased	N/A
			79	Female	MS, non‐obstructive CAD	28.9%	Discharged in good condition	NR
Park et al. (2020)^47^	Case series	Korea	78	Female	None	Severe LV systolic dysfunction	Deceased	N/A
			73	Female	None	Severe LV systolic dysfunction	Deceased	N/A
Meyer et al. (2020)^48^	Case report	Switzerland	83	Female	HTN	NR	Discharged in good condition	Only mild residual apical hypokinesis on the day of discharge
Eftekharzadeh et al. (2022)^49^	Case report	USA	94	Female	Anxiety disorder	NR	Deceased	N/A
Frynas‐Jończyk et al. (2022)^50^	Case report	Poland	76	Female	HTN, Asthma, DVT	30%	Discharged in good condition	On 2 months follow‐up: resolution of apical ballooning and improvement of EF to 58%
Fujiyoshi et al. (2022)^51^	Case report	Japan	71	Female	HTN, anxiety disorder	58%	Discharged in good condition	On 2 weeks follow‐up: normal LV wall motion with trivial apical hypertrophy and EF: 63%
Kimura et al. (2021)^52^	Case report	Japan	68	Female	HTN	50%	Discharged in good condition	On 66 days follow‐up: marked decrease in apical ballooning of the left ventricle, indicating good recovery
Mishra et al. (2021)^53^	Case report	USA	70	Male	HTN, DLP, DM, COPD, AF, status post cardioversion, ablation	NR	Deceased	N/A
Namburu et al. (2021)^54^	Case report	USA	69	Male	HTN	45%	Discharged in good condition	On week 1 follow‐up: improvement in LVEF (60%) with resolution of right atrial thrombus
Rivera et al. (2021)^55^	Case report	Spain	94	Female	HTN, paroxysmal AF, CVA	NR	Discharged in good condition	At 2 months follow‐up TTE demonstrated recovery of ventricular contractility
Wildermann et al. (2022)^56^	Case report	Germany	39	Female	MS	NR	Discharged in good condition	NR
Bapat et al. (2020)^57^	Case report	USA	67	Female	HTN, DM, asthma	61%	Discharged in good condition	NR
Chao et al. (2020)^58^	Case report	USA	49	Male	None	40%	Discharged in good condition	Normalization of LVEF to 55% and marked improvement in regional wall motion abnormalities
Dabbagh et al. (2020)^59^	Case report	USA	67	Female	Nonischemic cardiomyopathy	40%	Discharged in good condition	Stable ejection fraction and resolution of pericardial effusion
Manzur‐Sandoval et al. (2021)^60^	Case report	USA	54	Female	HTN, DM	NR	Discharged in good condition	Reversal of regional wall‐motion abnormalities in the apical two‐chamber view and in the left ventricular longitudinal strain
Sang et al. (2020)^61^	Case report	USA	58	Female	HTN, COPD, RA	Poor	Deceased	N/A
Tutor et al.(2021)^62^	Case series	USA	78	Male	HTN, CAD, CKD, AF	25%	Deceased	N/A
			76	Male	HTN, DM, DLP	30%	Discharged in good condition	4 months follow‐up: LVEF 55%–60% with no wall motion abnormality
Takotsubo after social stress articles								
Habedank et al. (2020)^63^	Case report	Germany	63	Female	HTN, anxiety, depression	35%	Discharged in good condition	On 4 days follow‐up: still moderate hypokinesia in the mid‐anterior section and LVEF recovered too normal
Giannitsi et al. (2020)^64^	Case report	Greece	79	Female	HTN	35%	Discharged in good condition	NR
Parker et al. (2020)^65^	Case report	Australia	69	Female	Lung cancer	34%	Discharged in good condition	NR
Uhe et al. (2020)^66^	Case report	Germany	81	Female	HTN, CKD	45%	Discharged in good condition	On 2 months follow‐up: full recovery of LV function
Chadha. (2020)^67^	Case report	USA	85	Female	None	35%	Discharged in good condition	On 5 days follow‐up: complete recovery of the LV systolic function
Rivers et al. (2020)^68^	Case report	Australia	71	Female	None	NR	Discharged in good condition	NR
Koutroumpakis et al. (2020)^69^	Case report	USA	65	Female	None	30%	Discharged in good condition	On 6 weeks follow‐up: normalization of the left ventricular function
Jabri et al. (2020)^5^	Cohort (N = 20 participants)	USA	Mean: 63	Female (*N* = 13), Male (*N* = 7)	DM (*N* = 3), HTN (*N* = 19), DLP (*N* = 14), CAD (*N* = 5), AF (*N* = 3), CKD (*N* = 2), COPD (*N* = 2)	30 (IQR: 25‐35)	Deceased (*N* = 1), discharged in good condition (*N* = 19)	NR
Dolci et al. (2021)^70^	Case report	Italy	65	Female	HTN, DLP	NR	Discharged in good condition	On 6 weeks follow‐up: patient was asymptomatic with full recovery of LV function
Moady et al. (2021)^71^	Case series	Israel	81	Female	DLP, hypothyroidism	38%	Discharged in good condition	On discharge: mild apical hypokinesia without left ventricular outflow tract obstruction (LVEF: 44%)
			70	Female	Hypothyroidism, AML	42%	Discharged in good condition	On discharge: normal cardiac anatomy and function
Kir et al. (2021)^9^	Case series	USA	85	Female	DM, HTN, DLP, CKD	45%–50%	Discharged in good condition	On 4 weeks follow‐up: complete resolution of her cardiomyopathy
			70	Female	HTN	30%	Discharged in good condition	On 1 month follow‐up: normal left ventricular wall motion, confirming her prior cardiomyopathy to be stress‐mediated (LVEF: 60%–65%)
Mohammed et al. (2020)^72^	Case report	USA	60	Female	DLP, anemia	22%	Discharged in good condition	NR
Ben Ammar et al. (2021)^73^	Case report	Tunis	59	Male	DM, HTN, DLP, CVA	40%	Discharged in good condition	NR
Takotsubo after COVID‐19 vaccination articles								
Vidula et al. (2021)^11^	Case series	USA	60	Female	CAD	44%	Discharged in good condition	NR
Boscolo Berto et al. (2021)^74^	Case report	Switzerland	63	Female	None	40%	Discharged in good condition	NR
Fearon et al. (2021)^75^	Case report	USA	73	Female	HTN, CKD, COPD, RA, asthma, hepatocellular carcinoma	20%	Discharged in good condition	On 3 days follow‐up: mild Improvement in biventricular function (LVEF: 35%–40%)
Crane et al. (2021)^76^	Case report	Australia	72	Male	DM, HTN, DLP, CABG, UC	38%	Discharged in good condition	On 5 days follow‐up: complete resolution of systolic dysfunction and wall motion abnormalities (EF: 52%)
Stewart et al. (2022)^77^	Case report	UK	Early 50s (52.5)	Female	COPD	NR	Discharged in good condition	Normal left ventricular (LV) systolic function and resolution of previously noted wall motion abnormalities
Tedeschi et al. (2022)^78^	Case report	Italy	71	Female	Congenital LQTS (mutation in KCHNQ 1 gene), catheter ablation for paroxysmal atrial fibrillation, mitral prolapse with mild mitral regurgitation	38%	Discharged in good condition	On Day 21 after the first dose: improvement of the LVEF up to 50%
Toida et al. (2022)^79^	Case report	Japan	80	Female	ESRD (renal sclerosis), HTN, secondary hyperparathyroidism with hyperphosphatemia	48%	Discharged in good condition	Normalized contractility of the apical myocardial segment, with the normalization of LV ejection fraction systolic function of 63%
Yamaura et al. (2022)^80^	Case report	Japan	30	Female	NR	NR	Discharged in good condition	On 15 day, The LV contraction had returned to normal range during follow‐up transthoracic Doppler echocardiography examination

Abbreviations: AF, atrial fibrillation; AML, acute myeloid leukemia; CAD, coronary artery disease; CKD, chronic kidney disease; COPD, chronic obstructive pulmonary disease; COVID‐19, coronavirus disease, 2019; CVA, cerebrovascular accident; DLP, dyslipidemia; DM, diabetes mellitus; DVT, deep vein thrombosis; ECG, electrocardiogram; ESRD, end‐stage renal disease; HTN, hypertension; LV, left ventricular; LVEF, left ventricular ejection fraction; MS, multiple sclerosis; NR, not reported; RA, rheumatoid arthritis; TTE, transthoracic echocardiogram; UC, ulcerative colitis.

Among patients with COVID‐19, the most common presenting clinical symptoms were dyspnea in 42/57 (73.6%), fever in 37/57 (64.9%), and cough in 29/57 (50.8%). However, among patients with emotional triggers, and patients with COVID‐19 vaccination, chest pain in 19/22 cases (86.3%) was the most common symptom (Table 2). In COVID‐19‐positive patients, the mean time interval from the first symptom to admission was 7.2 days (range 1–14 days), while it was 3.1 (range 0.08–10 days) in COVID‐19‐negative cases (Table 2). Out of 60 cases with COVID‐19, chest imaging, including chest X‐ray (CXR) or chest computed tomography scan, was performed in 45 cases. The most common chest imaging finding was ground‐glass opacity (GGO) which was reported in 14 (31.1%) participants (Table 2). During hospitalization, mechanical ventilation was required in 37/53 patients with COVID‐19 (69.8%). Of these patients, one was kept at continuous positive airway pressure and another used bilevel positive airway pressure. COVID‐19‐negative patients did not require oxygen support (Table 2). Among patients with COVID‐19, the most commonly reported in‐hospital complications were cardiogenic shock in 9/42 (21.4%), acute respiratory distress syndrome in 7/42 (16.6%), and acute kidney injury in 7/42 (16.6%). Among COVID‐19‐negative individuals, only two reported complications. One was cardiac arrest and the other was a gradual decline in the patient's visual acuity (Table 2).

**Table 2:** Baseline characteristics and clinical presentations of COVID‐19

Study, year	Clinical presentation	Time from symptom onset to admission	Heart rate (beats/min)	Blood pressure (mmHg)	Respiratory rate (breaths/min)	Saturation without O_2_ (%)	Saturation with O_2_ (%)	Temperature (°C)	Physical examination	Chest imaging	Oxygen support	In‐hospital complication
Takotsubo after COVID‐19												
Nguyen et al. (2020)^19^	Dyspnea	NR	75	119/76	NR	89%	100%	NR	NR	CT scan: ground glass opacity involving 10–20% of the lungs	Mechanical ventilation	NR
Panchal et al. (2020)^20^	Fever, dyspnea, cough, malaise	14 days	64	159/70	23	NR	87%	39.3	NR	CXR: multifocal pneumonia	Mechanical ventilation	Pulseless electrical arrest
Kariyanna et al. (2020)^21^	Cough, stroke presentation, right‐sided gaze, loss of appetite	4 days	98	146/97	32	NR	89%	37	NR	CXR: diffuse bilateral infiltrates	Mechanical ventilation	AKI, pulseless electrical arrest
Alizadehasl et al. (2022)^22^	Fever, dyspnea, chest pain, cough, diaphoresis	5 days	115	90/65	28	77%	NR	37.9	NR	CT scan: ground glass opacities compatible and congestion	Nasal mask	NR
Fujisaki et al. (2021)^23^	Fever, dyspnea	14 days	148	145/88	30	NR	75%	38.4	Bilateral crackles and tachycardia	CXR: diffuse opacities throughout the lung fields	Mechanical ventilation	ARDS, AKI, septic shock, cardiogenic shock
Demertzis et al. (2020)^24^	Fever, dyspnea, diarrhea, myalgia	2 days	NR	NR	NR	55%	NR	38.5	Bilateral crackles with end‐expiratory rhonchi, and systolic murmur along the left sternal border unchanged with respiration	CXR: bilateral multifocal patchy opacities	Mechanical ventilation	Cardiogenic shock
	Dyspnea, Orthopnea, cough	7 days	NR	NR	NR	NR	NR	NR	Bilateral crackles on auscultation, and muffled heart sounds	CXR: significant enlargement of the cardiac silhouette	NR	NR
Torabi et al. (2021)^25^	Fever, altered mental status	7 days	139	93/62	NR	NR	89%	38.2	Diffuse crackles and no cardiac murmurs	CXR: patchy consolidative opacities in the lung fields	Mechanical ventilation	Septic shock, cardiogenic shock
Ortuno et al. (2021)^26^	Fever, dyspnea, cough	5 days	NR	NR	NR	NR	93%	37.2	Bilateral diffuse crackling	CT scan: typical bilateral opacity	Mechanical ventilation	ARDS, AKI, cardiogenic shock
Hegde et al. (2020)^27^	Cough, myalgia	NR	NR	NR	NR	NR	NR	NR	NR	NR	Mechanical ventilation	AKI, shock, AF RVR
	Fever, altered mental status	NR	NR	NR	NR	NR	NR	NR	NR	NR	Mechanical ventilation	AKI
	Dyspnea	NR	NR	NR	NR	NR	NR	NR	NR	NR	Mechanical ventilation	ARDS, chronic respiratory failure
	Fever, dyspnea, cough	NR	NR	NR	NR	NR	NR	NR	NR	NR	Nasal mask	ARDS, shock
	Dyspnea, malaise	NR	NR	NR	NR	NR	NR	NR	NR	NR	Mechanical ventilation	Bilateral pleural effusion status post thoracentesis
	Dyspnea	NR	NR	NR	NR	NR	NR	NR	NR	NR	Mechanical ventilation	Bilateral pneumothorax status post chest tube placement, transient transaminitis
	Fever, dyspnea	NR	NR	NR	NR	NR	NR	NR	NR	NR	Mechanical ventilation	AKI, metabolic encephalopathy
Hoepler et al. (2021)^28^	Chest pain	NR	NR	NR	NR	NR	NR	NR	NR	CXR: unremarkable	Nasal mask	None
	Dyspnea	NR	NR	NR	NR	NR	NR	NR	Severely compromised regarding respiration	NR	Nasal mask	None
	NR	NR	NR	NR	NR	NR	NR	NR	NR	NR	Mechanical ventilation	Respiratory failure, cardiogenic shock, cardiac arrest
Alshamam et al. (2021)^4^	Dyspnea	1 day	Normal range	Normal range	30	50%	80%	Normal range	Respiratory distress, diffuse bilateral pulmonary crackles, mild jugular venous distention, and minimal bilateral pitting edema	CXR: diffuse bilateral opacification attributing to pneumonia and/or pulmonary edema	BiPAP	ARDS, AKI, Severe microcytic anemia
Bernardi et al. (2020)^29^	Fever, dyspnea	NR	95	135/85	NR	NR	NR	38	NR	CXR: diffuse hazy densities	CPAP	Cardiogenic shock
Sattar et al. (2020)^30^	Fever, cough, malaise, myalgia	14 days	114	133/64	24	92%	NR	36.9	Bilateral coarse crackles most prominent in the lower lung fields	CXR: bibasilar mixed ground glass opacities	Nasal mask	Atrial fibrillation
Tsao et al. (2020)^31^	Fever, cough, fatigue, myalgia	NR	NR	NR	NR	NR	NR	NR	NR		Mechanical ventilation	ARDS, vasodilatory shock
Gomez et al. (2020)^32^	Fever, dyspnea, cough, sore throat, rhinorrhea	5 days	89	118/70	18	93%	NR	39.7	Wheezes on pulmonary examination	CXR: diffuse bilateral alveolar infiltrates without cephalization or prominent pulmonary vascular markings	Mechanical ventilation	ARDS, cardiogenic shock, multiorgan failure
Belli et al. (2021)^18^	NR	NR	NR	NR	NR	NR	NR	N/A	NR	CT scan: right‐sided ground glass opacities and left‐sided dense ground glass with consolidation	Mechanical ventilation	NR
Titi et al. (2021)^33^	Fever, dyspnea, diarrhea	3 days	95	120/80	NR	95%	NR	37	Bilateral, basal crackles and decreased breath sounds	CT scan: lung ground glass opacities and subpleural patchy areas of consolidation	Mechanical ventilation	Cardiogenic shock, pericardial effusion
Faqihi et al. (2020)^34^	Chest pain, cough, myalgia	4 days	Normal range	Normal range	Normal range	Normal range	Normal range	Normal range	Mild tachypnea and decreased breath sound at the lung bases	CXR: interstitial infiltrates and consolidations	Mechanical ventilation	Cardiogenic shock
Solano, López et al. (2020)^35^	Fever, dyspnea, chest pain, cough	8 days	NR	SBP < 90	NR	NR	NR	N/A	NR	CXR: bilateral infiltrates, CT scan: perihilar ground‐glass opacities	NR	NR
Pasqualetto et al. (2020)^36^	Fever, dyspnea, chest pain, cough	10 days	NR	220/100	NR	NR	NR	N/A	NR	CT scan: ground‐glass opacities and bilateral consolidation in the lungs	Nasal mask	NR
	Fever, dyspnea, chest pain, cough	10 days	NR	NR	NR	NR	NR	N/A	NR	CT scan: ground‐glass opacities and bilateral consolidation in the lungs	Mechanical ventilation	None
	Fever, dyspnea, chest pain, cough	10 days	NR	NR	NR	NR	NR	N/A	NR	CT scan: ground‐glass opacities and bilateral consolidation in the lungs	Nasal mask	NR
Koh et al. (2021)^37^	Fever, dyspnea, chest pain, cough, diarrhea, nausea, and vomiting	2 days	125	82/45	30	95%	NR	39.4	NR	NR	Mechanical ventilation	NR
Dave et al. (2020)^38^	Fever, dyspnea, myalgia, diarrhea	5 days	Normal range	Normal range	Tachypnea	Normal range	NR	Normal range	NR	CXR: right middle and lower lung infiltrates	Mechanical ventilation	None
Van Osch et al. (2020)^39^	Fever, dyspnea	NR	70	150/70	25	92%	NR	N/A	Bilateral inspiratory crackles and expiratory rhonchi	CXR: bilateral consolidations	Mechanical ventilation	NR
Bhattacharyya et al. (2020)^40^	Dyspnea	3 days	Normal range	150/100	Normal range	Normal range	NR	Normal range	NR	NR	NR	NR
Taza et al. (2020)^41^	Fever, dyspnea	NR	Normal range	NR	Tachypnea	hypoxic	NR	Febrile	NR	NR	Mechanical ventilation	NR
Bottiroli et al. (2020)^42^	Fever, cough	9 days	81	135/65	22	NR	90%	38.2	NR	CXR: diffuse opacities mainly in the right lung, CT scan: diffuse bilateral ground glass opacities	Mechanical ventilation	NR
Roca et al. (2020)^43^	Fever, dyspnea, cough, fatigue	14 days	Tachycardia	NR	NR	NR	91%	N/A	NR	CXR: multiple patchy shadows in both lungs and parenchymal thickening with bilateral basal alveolar interstitial infiltrates	Nasal mask	NR
Oyarzabal et al. (2020)^44^	Chest pain	NR	NR	NR	NR	NR	NR	N/A	NR	CXR: unremarkable	NR	NR
Minhas et al. (2020)^45^	Fever, cough, fatigue, diarrhea	5 days	130	156/95	24	NR	82%	38.7	Diffuse rhonchi	CXR: lower lobe predominant bilateral infiltrates	NR	NR
Kong et al. (2021)^46^	Fever, fatigue, loss of appetite	Several days	Tachycardia	NR	NR	NR	NR	N/A	NR	NR	Mechanical ventilation	None
	NR	NR	Tachycardia	NR	NR	hypoxic	NR	39.1	NR	NR	Mechanical ventilation	NR
Park et al. (2020)^47^	Fever, dyspnea, sore throat	7 days	112	114/76	24	60	NR	38.4	NR	CXR: diffuse infiltration of whole lung fields	Mechanical ventilation	None
	Fever, cough	13 days	70	148/78	28	NR	NR	37.6	NR	CXR: typical diffuse ground‐glass appearance, CT scan: diffuse infiltration of bilateral lung fields	Mechanical ventilation	None
Meyer et al. (2020)^48^	Fever, dyspnea, chest pain, cough	3 days	Normal range	Normal range	Normal range	Normal range	NR	Normal range	Normal	CXR: clear bilateral lung opacities	Nasal mask	NR
Eftekharzadeh et al. (2022)^49^	Dyspnea	7 days	118	196/93	46	70%	96%	37.2	Accessory muscle use with rales and rhonchi	CXR: clear lungs with cardiomegaly	None	None
Frynas‐Jończyk et al. (2022)^50^	Fever, cough, dyspnea	14 days	60	134/76	NR	95%	NR	NR	NR	CT scan: multiple peripheral, small areas of ground‐glass opacities	None	None
Fujiyoshi et al. (2022)^51^	Fever, dyspnea	NR	NR	NR	NR	NR	NR	NR	NR	CT scan: trivial peripheral consolidations	None	None
Kimura et al. (2021)^52^	Dysarthria, gait disturbance, fever, cough	14 days	120	152/114	NR	NR	NR	33.2	NR	CT scan: bilateral pneumonia	Mechanical ventilation	None
Mishra et al. (2021)^53^	Dyspnea, fever	7 days	88	146/64	28	NR	85%	NR	Reduced breath sounds bilaterally, along with mild infrascapular crackles	CT scan: bilateral ground glass opacities and infiltrates	NR	None
Namburu et al. (2021)^54^	Chest pain, dyspnea	7 days	124	132/88	28	83%	95%	37.1	Moderate to severe respiratory distress with bilateral basilar crackles and sinus tachycardia	CXR: bilateral patchy opacities most prominent at left lung base with minimal left pleural effusion	None	Bilateral pulmonary embolism with right heart strain
Rivera et al. (2020)^55^	Dyspnea, cough	2 days	NR	NR	NR	NR	NR	NR	NR	CXR: bilateral basal pneumonia	None	None
Wildermann et al. (2022)^56^	Malaise, fatigue, nystagmus, dizziness, headache, cough, dyspnea	10 days	NR	NR	NR	NR	NR	NR	NR	CT scan: multifocal central and peripheral ground glass opacities involving both pulmonary lobes	NR	None
Bapat et al. (2020)^57^	Dyspnea, nausea	4 days	118	NR	NR	<90%	NR	NR	NR	CXR: bilateral predominantly peripherally distributed patchy opacities	Mechanical ventilation	None
Chao et al. (2020)^58^	Fever, cough	NR	NR	NR	NR	NR	NR	NR	NR	CXR: severe diffuse bilateral pulmonary infiltrates consistent with ARDS	Mechanical ventilation	None
Dabbagh et al. (2020)^59^	Cough, dyspnea, and left shoulder pain	NR	122	118/82	24	Normal	Normal	36.8	Normal	Unremarkable	None	None
Manzur‐Sandoval et al. (2021)^60^	Cough, fever, dyspnea	3 days	75	100/60	NR	82%	NR	NR	Diffuse pulmonary rales	CXR: bilateral diffuse interstitial infiltrates	Mechanical ventilation	None
Sang et al. (2020)^61^	Fever, dyspnea	NR	>200	NR	NR	NR	NR	NR	NR	CXR: bronchiectasis with interstitial thickening	Mechanical ventilation	None
Tutor et al. (2021)^62^	Confusion, dyspnea	NR	Tachycardia	90/50	20	85%	NR	39	NR	CXR: bilateral pulmonary infiltrates	Mechanical ventilation	Multiorgan failure
	Cough, dyspnea, fever	NR	Tachycardia	Normal	14	90%	NR	Normal	NR	CXR: bilateral multifocal infiltrates	Nasal mask	NR
Takotsubo after social stress												
Habedank et al. (2020)^63^	Chest pain	NR	NR	NR	NR	NR	NR	NR	NR	NR	None	Cardiac arrest
Giannitsi et al. (2020)^64^	Chest pain	NR	75	130/70	NR	99	NR	NR	Normal	NR	None	None
Parker et al. (2020)^65^	Chest pain	NR	NR	NR	NR	NR	NR	NR	NR	NR	None	None
Uhe et al. (2020)^66^	Dyspnea, chest pain	NR	78	130/73	NR	NR	NR	37.2	NR	NR	None	None
Chadha et al. (2020)^67^	Chest pain	NR	NR	NR	NR	NR	NR	NR	Normal	CXR: unremarkable	None	None
Rivers et al. (2020)^68^	Chest pain	NR	NR	NR	NR	NR	NR	NR	NR	NR	None	None
Koutroumpakis et al. (2020)^69^	Chest pain, diaphoresis	NR	63	142/77	NR	NR	NR	NR	Normal	NR	None	None
Jabri et al. (2020)^5^	NR	NR	NR	NR	NR	NR	NR	NR	NR	NR	NR	NR
Dolci et al. (2020)^70^	Chest pain	NR	NR	NR	NR	NR	NR	NR	NR	NR	None	None
Moady et al. (2021)^71^	Chest pain	2 days	100	100/60	NR	NR	NR	NR	Apical systolic heart murmur with no signs of heart failure	NR	None	None
	Chest pain	Few hours	95	170/80	NR	NR	NR	NR	Normal	NR	None	None
Kir et al. (2021)^9^	Dyspnea, chest pain	2 h	120	108/45	15	95	NR	36.9	Tachycardic, with normal pulses, normal cardiac exam with no significant murmur or rub on auscultation, lungs were clear to auscultation	NR	None	None
	Dyspnea, chest pain, diaphoresis	1 day	NR	NR	NR	NR	NR	NR	NR	NR	None	Gradual decline in her visual acuity
Mohammed et al. (2020)^72^	Dyspnea, chest pain, diaphoresis, disorientation	7 days	115	188/82	NR	NR	NR	NR	NR	CXR: unremarkable	None	None
Ben Ammar et al. (2021)^73^	Weird behavior, psychomotor agitation	NR	100	140/80	NR	99%	NR	37	NR	NR	None	None
Takotsubo after COVID‐19 vaccination articles												
Vidula et al. (2021)^11^	Chest pain	4 days	NR	NR	NR	NR	NR	NR	NR	NR	None	None
Boscolo Berto et al. (2021)^74^	Fever, dyspnea	1 day	NR	NR	NR	NR	NR	NR	NR	CT scan: revealed no pulmonary embolism but did show signs of heart failure	None	None
Fearon et al. (2021)^75^	Dyspnea, chest pain	17 h	118	108/57	24	Normal range	NR	Normal range	Jugular venous distention	NR	None	None
Crane et al. (2021)^76^	Dyspnea, chest pain	3 days	99	130/65	Tachypnoea	Normal	NR	36.1	Normal	CT angiography: excluded any pulmonary embolism	None	None
Stewart et al., 2021^77^	Chest pain, diaphoresis, dyspnea, vomiting	NR	NR but normal	NR but normal	NR but normal	NR but normal	NR but normal	NR but normal	NR but normal	Chest radiograph: absence of consolidation or pleural effusion	None	None
Tedeschi et al. (2022)^78^	Chest pain, dyspnea	10 days	NR	NR	NR	93%	NR	37.2	Bilateral diffuse crackles	NR	None	None
Toida et al. (2022)^79^	Hypotension during dialysis	3 days	114	82/47	NR	NR	NR	NR	Systolic murmur of Levine 2/6 at the second left sternal border, clear lung sounds, and no leg edema	NR	None	None
Yamaura et al. (2023)^80^	Chest pain, diaphoresis	Few hours	NR	NR	NR	NR	NR	NR	NR	NR	None	None

Abbreviations: AF, atrial fibrillation; AKI, acute kidney injury; ARDS, acute respiratory distress syndrome; BiPAP, bilevel positive airway pressure; COVID‐19: coronavirus disease, 2019, CPAP, continuous positive airway pressure; CT, computed tomography; CXR, chest X‐ray; ECG: electrocardiogram, N/A, not/applicable; NR, not reported; RVR, rapid ventricular rate.

Electrocardiogram (ECG) findings were reported in 81 cases. The most common abnormalities were T‐wave inversion in 35 (43.2%), ST‐segment elevation in 30 (37%) and QT interval prolongation in 13 (16%). In addition, atrial fibrillation was reported in 6 (7.4%). Out of 82 cases, apical ballooning was reported in 31 (37.8%) (Table 3). Regarding cardiac biomarkers, BNP was measured in 38 cases and was found to be elevated in 36 (94.7%). Troponin was measured in 74 cases and was found to have been raised in 71 (95.9%). Also, CK was measured in 28 cases and found to be raised in 14 (50%) (Table 3).

**Table 3:** Cardiac laboratory and paraclinical findings

Study, year	ECG findings	TTE findings	CA findings	Troponin elevation	BNP elevation	Creatine kinase elevation
Takotsubo after COVID‐19						
Nguyen et al. (2020)^19^	Sinus rhythm with prolonged QT interval (QTc 521 ms)	NR	Significant lesions on the proximal LAD and the first diagonal arteries. The ventriculogram showed regional wall motion abnormality unrelated to the coronary lesions, compatible with a median takotsubo.	Positive	NR	NR
Panchal et al. (2020)^20^	Nonspecific ST‐T wave abnormality (QTc 420 ms, QTc 439 ms on admission)	New left ventricular (LV) regional wall motion abnormality with hypokinesis of the basal to midsegments and hyperkinetic apical segments	NR	NR	NR	NR
Kariyanna et al. (2020)^21^	Normal range sinus rhythm, Q waves in V1–V2 leads and Q waves with ST segment elevation V3, V4, V5 and deep T wave inversion in V6	Diffuse hypokinesis with distinct regional wall motion abnormality, apical dyskinesis or apical systolic ballooning suggestive of stress induce cardiomyopathy	NR	Positive	Positive	NR
Alizadehasl et al. (2022)^22^	Sinus tachycardia with mild dynamic ST segment depression in precordial leads	Akinesia in mid‐to‐apical segments of both ventricles compatible with biventricular apical ballooning syndrome	NR	Positive	NR	NR
Fujisaki et al. (2021)^23^	Atrial fibrillation, poor R progression, and negative T waves in lead I, aVL, and V2–V6	Severe hypokinetic biventricular apical and mid segments	NR	Positive	Positive	NR
Demertzis et al. (2020)^24^	Normal range sinus rhythm, QTc 387 ms	New reduced EF (40%) and severe hypokinesis of the basal–mid inferoseptal, inferior, anteroseptal, and anterior walls	NR	Positive	NR	Positive
	Normal sinus rhythm, QTc 427 ms	Large pericardial effusion with signs of right ventricular dysfunction and apical hypokinesis	NR	Positive	Positive	NR
Torabi et al. (2021)^25^	Low voltage in the limb leads	Hyperdynamic left ventricle and a hemodynamically significant moderate‐sized pericardial effusion with right atrial systolic collapse. The LV apex was dilated with systolic hypokinesis and basal segments had preserved contraction	Normal range coronaries and mildly elevated left ventricular end diastolic pressure	Positive	Positive	NR
Ortuno et al. (2021)^26^	Non‐elevated ST segment, prolonged QT interval, T wave inversion	Left ventricular failure with reduced ejection fraction (LVEF 40%) and typical apical ballooning suggesting	Positive	NR	NR	NR
Hegde et al. (2020)^27^	Atrial flutter RVR with diffuse ST elevations	Left ventricular EF: 15%	NR	Positive	Positive	NR
	Atrial fibrillation, with RVR, diffuse deep T‐wave inversions	Left ventricular EF: 53%	NR	Positive	Positive	Positive
	Sinus rhythm with diffuse ST‐T changes	Left ventricular EF: 45%	NR	Negative	Negative	Negative
	Sinus rhythm with deep T‐wave inversions	Left ventricular EF: 20%	NR	Positive	Positive	Negative
	Atrial fibrillation, with diffuse ST‐T changes	Left ventricular EF: 30%	NR	Positive	Positive	Negative
	Sinus tachycardia with PACs and T‐wave inversions	Left ventricular EF: 40%	NR	Positive	Negative	Negative
	Sinus tachycardia with diffuse ST‐T changes	Left ventricular EF: 45%	NR	Positive	Positive	Positive
Hoepler et al. (2021)^28^	Complete right bundle branch block (QRS 160 ms) with T wave inversions in leads I, aVL, and V3–V6. Three days later, the QRS complex was normal range again but T wave inversions were more pronounced	Severe hypo‐ to akinesia in parts of the apical and the inferoapical wall with hypercontractility of the basal segments of the heart	Normal‐range coronary arteries	Positive	NR	Negative
	ST‐elevations and negative T waves in leads V4 and V5 and isolated negative T waves in leads II, III, aVF, and V6 (the patient later developed persistent T wave inversions in II, III, aVF, and V2 to V6)	Moderately reduced systolic function and apical, anterior, and posterolateral akinesia	Severe three‐vessel coronary artery disease (CAD), but also TT cardiomyopathy with classic apical ballooning and hyperkinesia of the basal segments	Positive	Positive	Positive
	Hyperacute T waves, which were later replaced by deep T wave inversions in V3 to V6	Severe apical akinesia with hyperkinesia of the basal segments and a minimum ejection fraction (EF) of 20% after it had been only moderately reduced 2 days earlier	Positive	Positive	Positive	Negative
Alshamam et al. (2021)^4^	ST‐segment elevation in leads V1–V5 and T‐wave inversions in leads I and aVL	Mid to apical left ventricular (LV) akinesia with preserved function in the proximal and segment, aortic valve sclerosis, reduced excursion of Trileaflet valve (without stenosis), and mild‐to‐moderate tricuspid regurgitation with moderate pulmonary artery systolic pressure (PASP)	NR	Positive	Positive	Positive
Bernardi et al. (2020)^29^	ST‐segment elevation in anterolateral leads, suggesting an acute myocardial infarction	Dilated left ventricle with akinesis of the mid and apical ventricle segments with hyperkinesis of the basal segments and severe systolic dysfunction (left ventricle ejection fraction calculated by Simpson's biplane method [LVEF]: 30%); first‐grade diastolic dysfunction; partial left ventricle outflow tract obstruction determining a late maximal gradient of 56 mmHg with systolic anterior motion of the mitral valve and associated moderate to severe mitral regurgitation; and, finally, 2 large apical thrombotic formations: the positive terior one was elongated (maximum: 31 mm) and mobile, and the anterior one was wide and oval	Positive	Positive	Positive	Negative
Sattar et al. (2020)^30^	Atrial fibrillation with a rapid ventricular response, right bundle branch block (RBBB), and T‐wave inversions in the inferolateral leads	Left ventricle ejection fraction (LVEF) of 30% with diffuse anterior wall and apical akinesia and apical ballooning	Positive	NR	NR	NR
Tsao et al. (2020)^31^	Slight ST‐segment elevations diffusely with nonspecific T‐wave inversions	Severe hypokinesis of the mid‐left ventricular cavity, with normal range‐to‐hyperdynamic contractility of basal and apical left ventricular segments and a moderately reduced biplane ejection fraction of 36%	Positive	Negative	Positive	Negative
Gomez et al. (2020)^32^	Sinus tachycardia without ST‐wave or T‐wave changes, prolonged QTc interval of 516 ms and low‐voltage QRS in the precordial leads	Depressed left ventricular ejection fraction of 25%–30%, with severe hypokinesis of the mid‐to‐apical segments and preserved basal myocardial function	Positive	Positive	Positive	Negative
Belli et al. (2021)^18^	ST elevation with biphasic T waves and Q waves	Complete apical ballooning and extensive akinesia spanning multiple coronary territories with a global LV systolic function impairment	Nonsignificant 30% stenosis of the left anterior descending coronary artery with otherwise smooth coronary arteries	Positive	Positive	NR
Titi et al. (2020)^33^	Diffuse ST segment elevation, more evident in the precordial leads (V3–V5), and Q waves in precordial and peripheral inferior leads	Severe global reduction of the left ventricular contractility with mild pericardial effusion	70% stenosis in the posterolateral branch which originated from the circumflex (left dominance)	NR	NR	NR
Faqihi et al. (2020)^34^	Sinus tachycardia (115 beats/min) and nonspecific ST‐segment and T‐wave abnormalities in the precordial leads	LV basal and midventricular akinesia with apical sparing	NR	Positive	NR	Positive
Solano, López et al. (2020)^35^	2 mm ST elevation Inf and Lat lids	Akinesia of all basal segments	Normal range coronary arteries, left ventricular angiography presented basal segment akinesia and hypercontractility of the mid‐apical segments with elevated diastolic pressure	Positive	Positive	NR
Pasqualetto et al. (2020)^36^	Diffuse negative T waves on precordial leads with QT interval prolongation	Dyskinesia of the left ventricle apex (apical ballooning) and basal wall hypercontractility with systolic dysfunction, global preserved left ventricular ejection fraction (EF) of 53%	The autopsy confirmed a normal‐range coronary anatomy.	Positive	Positive	NR
	Diffuse negative T waves on precordial leads with QT interval prolongation	Dyskinesia of the left ventricle apex (apical ballooning) and basal wall hypercontractility with systolic dysfunction, LVEF (30%)	Negative for significant coronary stenosis	Positive	Positive	NR
	Diffuse negative T waves on precordial leads with QT interval prolongation	Dyskinesia of the left ventricle apex (apical ballooning) and basal wall hypercontractility with systolic dysfunction, moderately impaired LVEF (42%)	Negative for significant coronary stenosis	Positive	Positive	NR
Koh et al. (2021)^37^	There were diffuse ST‐segment elevations and PR‐segment depressions in the inferolateral leads. There were also ST‐segment depressions and PR‐ segment elevations in leads V1, aVR	Biventricular systolic dysfunction with a left ventricular ejection fraction (LVEF) of around 30%. This demonstrated global left ventricular hypokinesia (biplane LVEF 32.7% with average left ventricular [LV] global longitudinal strain of −8.7%). There was moderate right ventricular systolic dysfunction with a tricuspid annular plane systolic excursion of 11.7 mm, estimated pulmonary artery systolic pressure of 42 mmHg, borderline pulmonary artery acceleration time (120 ms) and echocardiographic estimated pulmonary vascular resistance of 3.41 wood units. A small pericardial effusion was also found. CMR showed an improved LVEF of 66%. There was maximal LV wall thickness of 8 mm at the basal anteroseptal segment, normal range right ventricular systolic function and indexed volumes and there was no late gadolinium enhancement (LGE) in the myocardium of both ventricles or myocardial edema	Normal range epicardial vessels with slow coronary flow. Left ventriculography revealed global left ventricular hypokinesia with severe left ventricular systolic dysfunction	Positive	NR	NR
Dave et al. (2020)^38^	Sinus tachycardia and nonspecific T‐wave abnormality in the lateral leads	Normal range right ventricular function, left ventricular ejection fraction (LVEF) 26% with preserved basal function, and apical ballooning consistent with takotsubo cardiomyopathy	NR	Positive	Positive	NR
Van Osch et al. (2020)^39^	Negative T‐waves were observed at the monitor and a 12‐lead ECG was obtained which showed sinus rhythm with diffuse, new, deeply negative T‐waves and a prolonged QTc interval of 505 ms	A poor left ventricular systolic function [left ventricular ejection fraction (LVEF) approximately 30%] with circumferential akinesia of the apex in the mid‐ventricular and apical segments and circumferential hyperdynamic contractions of the basal segments consistent with the diagnosis takotsubo cardiomyopathy	Low calcium score and a nonsignificant stenosis (<50%) in the proximal left anterior descending	Positive	NR	NR
Bhattacharyya et al. (2020)^40^	Inferolateral ST‐segment elevation	Hypokinetic mid and akinetic apical left ventricular (LV) segments and hypercontractile basal segments with prominent apical ballooning typical of takotsubo cardiomyopathy (TTC). Two‐dimensional speckle tracking echocardiography revealed LV global longitudinal strain (GLS) of −13.9 and ejection fraction (EF%) of 38%	Non‐obstructive coronary artery disease (CAD) involving the left anterior descending artery	Positive	Positive	NR
Taza et al. (2020)^41^	ST segment elevations in the inferior leads (II, III, aVF)	NR	Non‐obstructive coronary arteries and apical ballooning on ventriculography, consistent with takotsubo syndrome	Negative	NR	NR
Bottiroli et al. (2020)^42^	ST‐segment elevation with loss of R‐waves in leads V2 to V4	Normal range size of ventricular chambers, severe left ventricular (LV) systolic dysfunction with an LV ejection fraction (EF) of 25%, and akinesia of middle and apical segments (apex ballooning) with hyperkinetic motion of basal segments	NR	Positive	Positive	NR
Roca et al. (2020)^43^	The electrocardiogram showed negative T waves and repolarization phase alterations	Alterations in the left ventricle: apical akinetic expansion (apical ballooning) and hypokinesia of the mid‐ventricular segments with slightly reduced systolic function (ejection fraction slightly reduced to 48%)	NR	Positive	NR	Positive
Oyarzabal et al. (2020)^44^	The electrocardiogram showed a 1 mm ST segment elevation in leads V2–V3 and DI‐AVL	The findings of ventriculography were confirmed by echocardiography	Coronary angiography showed coronary arteries free of lesions and cardiac ventriculography was performed. This showed a very reduced left ventricular ejection fraction with extensive apical akinesia	NR	NR	NR
Minhas et al. (2020)^45^	Sinus tachycardia and 1‐mm upsloping ST‐segment elevations in leads I and aVL, mild diffuse PR interval depressions, and diffuse ST‐T wave changes	Akinetic middle to distal anterior, anteroseptal, antero‐lateral, and apical segments, moderately hypokinetic middle and distal inferolateral segments, and hyper‐dynamic basal segments. Apical ballooning was also noted. Left ventricular (LV) ejection fraction was 20%. The distal third or apical right ventricular (RV) free wall was akinetic, with hyperdynamic RV basal wall motion. RV function was mildly reduced	NR	Positive	NR	NR
Kong et al. (2021)^46^	Anteroseptal ST‐segment elevations	NR	Mild non‐obstructive coronary artery disease, and a left ventriculogram was performed which demonstrated preserved basal function with apical akinesis, consistent with TTS	Positive	NR	NR
	New ST‐segment elevations in the anterolateral leads	Apical hypokinesis, consistent with ventriculogram findings	Non‐obstructive coronary artery disease. A left ventriculogram demonstrated significantly reduced ejection fraction with preserved basal function and apical ballooning and akinesis, consistent with TTS. Right heart catheterization showed elevated biventricular filling pressures with reduction in CO and CI	Positive	NR	NR
Park et al. (2020)^47^	T wave inversion appeared	Apical ballooning with severe LV systolic function	NR	Positive	Positive	NR
	NR	Apical ballooning with dyskinetic movement and severe LV systolic dysfunction	NR	Positive	NR	Positive
Meyer et al. (2020)^48^	<1 mm ST‐segment elevation in all precordialleads with deep T‐wave inversions	Typical left ventricular apical ballooning with hyperkinetic basal segments	Nonsignificant lesions with a typical takotsubo syndrome (TTS) image on ventriculography	Positive	NR	NR
Eftekharzadeh et al. (2022)^49^	Tachycardia and ST‐segment elevation in inferior lateral leads II, III, aVF, and V5	NR	A 40% proximal to mid‐left anterior descending (LAD) lesion without any severe obstruction, moderate left ventricular (LV) dysfunction with apical ballooning was noted during the left heart chamber assessment	Positive	Positive	NR
Frynas‐Jończyk et al. (2022)^50^	Sinus rhythm of 80/min, left anterior fascicular block, ST‐segment depression in V1‐V5 leads, and negative T waves in II, III, aVF, V1–V6 leads	Apical dyskinesis resulting in apical ballooning and hypo‐akinesia of the mid‐ventricular segments with severely reduced left ventricular ejection fraction (LVEF) of 30%	Mild, non‐obstructive atherosclerotic plaques in the coronary arteries	Positive	Positive	Negative
Fujiyoshi et al. (2022)^51^	Deep T‐wave inversions in all precordial leads	Hypokinesis with hypertrophy in the apical region and hyperkinesis in the basal region with estimated LV ejection fraction of 58%	NR	Positive	NR	NR
Kimura et al. (2021)^52^	Inverted T‐waves in leads I, II, aVF, and V1–V6	Apical akinesis with preserved basal function and a depressed ejection fraction of around 50%	NR	Positive	Positive	Positive
Mishra et al. (2021)^53^	New onset T wave inversion across V1–V6	Hypokinesis of the basal region of the left ventricle with hyperkinesis of the apical region of the left ventricle consistent with a reverse takotsubo cardiomyopathy	NR	NR	NR	NR
Namburu et al. (2021)^54^	ST elevations in V1–V3 leads consistent with the diagnosis of ST‐elevation myocardial infarction (STEMI)	Left ventricular ejection fraction (LVEF) of 45%, enlarged right ventricle (RV), and a right atrial thrombus	Non‐obstructive coronary artery disease with apical ballooning of the left ventricle, a ventriculogram characteristic of TTC	Positive	NR	NR
Rivera et al. (2020)^55^	Atrial fibrillation with rapid ventricular response and ST‐segment elevation in the anterolateral leads	NR	Absence of obstructive coronary lesions and ventriculography showed severe ventricular dysfunction with anterolateral, apical, and inferior dyskinesia and hypercontractility of the basal segments, compatible with takotsubo syndrome (TTS)	NR	NR	NR
Wildermann et al. (2022)^56^	Intermittent ventricular bigeminy	NR	Cardiac catheterization showed no coronary artery disease but regional wall motion abnormalities compatible with atypical TTC	Positive	NR	NR
Bapat et al. (2020)^57^	Persistence of T wave inversions and progressive prolongation in the QT interval	Preserved left ventricular ejection fraction of 61% but with new apical hypokinesis	Not performed	Positive	NR	NR
Chao et al. (2020)^58^	Narrow QRS, precordial T‐wave inversion, QTc of 467 ms 3 days later: right bundle branch block, prolonged QTc of 539 ms, and mild diffuse ST elevation	Mild to moderately reduced LVEF of 40% with marked hypokinesis of basal and mid segments and pre‐served wall motion of apical segments	Not performed	Positive	Positive	Positive
Dabbagh et al. (2020)^59^	Low voltage in the limb leads with nonspecific ST‐segment changes serial ECG revealed deep T‐wave inversions in precordial leads (V2–V6)	Large pericardial effusion circumferentially around the entire heart with signs of early right ventricular diastolic collapse, dilated but collapsing inferior vena cava, and mitral valve inflow variation of 31% on pulsed wave Doppler. LVEF was mildly reduced at 40%, with no regional wall motion abnormalities, similar to TTE 1 year prior. Serial TTE demonstrated resolution of the pericardial effusion; however, the patient was found to have new hypokinesis of the apical and periapical walls concerning for takotsubo cardiomyopathy (TTC)	Not performed	Positive	NR	NR
Manzur‐Sandoval et al. (2021)^60^	Pulse rate 75 beats/min; PR interval 160 ms; QRS interval 100 ms; prolonged QTc interval 551 ms; QRS axis –30°; poor R‐wave progression; giant inverted T waves at V2– V6, DI, and AVL; and Q waves at DII and AVF.	In the apical 2‐chamber view, apical ballooning with normal contraction of the basal segments was observed; left ventricular longitudinal strain was decreased in the mid and apical segments	Not performed	Positive	Positive	Positive
Sang et al. (2020)^61^	Septal infarction pattern	Severely reduced left ventricular systolic function with global hypokinesis of the left ventricle. The apical segments had disproportionately poor function compared with the basal segments, a finding consistent with stress‐induced (takotsubo) cardiomyopathy	Not performed	Positive	Positive	NR
Tutor et al. (2021)^62^	Nonspecific ST‐T wave changes	Depressed LVEF of 25% with basal‐sparing and severe apical akinesis	NR	Positive	NR	Negative
	Diffuse t‐wave inversions	Severely depressed LV systolic function, and global hypokinesis with akinesis of the apex and basal sparing	NR	Positive	NR	Positive
Takotsubo after social stress						
Habedank et al. (2020)^63^	ST elevations 0.4 mV from J‐point in leads V2 to V4, 0.1 mV in lead aVL, and a QTc = 522 ms by Bazett's resp. 477 ms by Fridericia's formula	Moderate hypokinesia in the mid‐anterior section and cardiac MRI proving significant edema in the entire anterior and septal wall. severe hypokinesia in the mid‐apical segments and hyperdynamic basal segments	Moderate coronary sclerosis	Positive	NR	Negative
Giannitsi et al. (2020)^64^	Diffuse ST segment elevation	NR	Excluded stenotic lesions	Positive	NR	NR
Parker et al. (2020)^65^	Q waves and ST elevation in the inferior leads	NR	Chronic occlusion of right coronary artery, left ventricular ejection fraction of 34%, and basal hyperkinesis with mid‐ventricular and apical dyskinesis	Positive	NR	NR
Uhe et al. (2020)^66^	Negative T‐waves in II, III, aVF, and V3‐6	Wall motion abnormalities with apical septal dyskinesis, mid ubiquitous akinesia and basal septal and anterior hypokinesis. Left ventricular ejection fraction was reduced to 45%. Global longitudinal strain with a typical strain pattern of apical ballooning was 8%	No signs of artery disease (CAD)	Positive	NR	Positive
Chadha et al. (2020)^67^	A septal q‐ST pattern in leads V1–V3	Basal hyperkinesis and apical ballooning	Nonsignificant coronary artery disease	Positive	NR	NR
Rivers et al. (2020)^68^	Diffuse ST elevation	A dilated left ventricle with an akinetic apex and preserved contraction of the basal segments	No obstructive lesions	Positive	NR	NR
Koutroumpakis et al. (2020)^69^	A sinus rhythm at 63 beats/min, with marked T wave inversion in the inferior and anterolateral leads	Left ventricular systolic dysfunction with apical ballooning. basal hyperkinesis with dyskinesis of the apex	TIMI 2 flow down the left anterior descending artery	Positive	NR	NR
Jabri et al. (2020)^5^	NR	NR	NR	NR	NR	NR
Dolci et al. (2020)^70^	Regular sinus rhythm with nonspecific ST‐segment alterations in the inferior leads	Hypokinesia of the left ventricle (LV) mid segments with normal range apical and basal contraction resulting in mild reduction of LV ejection fraction. Normal range epicardial coronary arteries and confirmed mid‐ventricular ballooning with Normal range contraction of basal and apical segments	Normal range epicardial coronary arteries and confirmed mid‐ventricular ballooning with Normal range contraction of basal and apical segments	Positive	NR	NR
Moady et al. (2021)^71^	Normal sinus rhythm with diffuse ST segment elevation, most prominent in the anterior leads with no reciprocal changes	Moderately reduced global systolic left ventricular function with a typical pattern of apical ballooning and left ventricular outflow obstruction	Basal hypercontractility and apical ballooning were obvious during left ventriculography	Positive	Positive	NR
	Normal sinus rhythm with anterior ST segment elevation	Reduced apical contraction with estimated ejection fraction of 42% and hyperkinetic basal segments of the left ventricle	Normal arteries	Positive	Positive	NR
Kir et al. (2021)^9^	Sinus tachycardia with inferior Q waves, poor R wave progression, and nonspecific ST‐segment changes	Basal hyperkinesis with severe apical hypokinesis. An ejection fraction of 45%–50%, with severe hypokinesis of the apical segments with apical ballooning and basal hyperkinesis	Negative for any significant obstructive coronary artery disease	Positive	Positive	NR
	New deep T wave inversions in the precordial leads and subtle ST elevation in the inferior leads	Severe hypokinesis of the antero‐apical wall, concerning for anterior myocardial infarction. severely depressed left ventricular ejection fraction of 30% with antero‐apical and infero‐apical wall akinesis	Mild calcification and nonobstructive disease in the coronary arteries with a right dominant circulation. Myocardial bridge was noted in the proximal mid left anterior descending artery. Left ventricular end‐diastolic pressure was elevated at 22 mmHg.	NR	NR	NR
Mohammed et al. (2020)^72^	Sinus tachycardia and left bundle branch block	A newly depressed ejection fraction (EF) of 22%, a moderately increased left ventricular (LV) cavity size, and moderate hypokinesis of the mid‐distal left ventricular wall with preservation of basal LV contractility	Normal coronary arteries	Positive	Positive	NR
Ben Ammar et al. (2021)^73^	Elevated ST‐segment in leads V3 to V6	Reduced left ventricular ejection fraction which was 40%, There was also a decrease in the global longitudinal strain with a marked decrease in the apical segments	Severe multivessel disease, tight stenosis in the posterior‐right coronary artery. On the left, there were insignificant lesions in the mid and distal left anterior descending arteries, as well as insignificant lesions in the mid‐circumflex and second obtuse marginal arteries.	Positive	NR	NR
Takotsubo after COVID‐19 vaccination						
Vidula et al. (2021)^11^	Inferolateral T wave inversions	Mildly reduced LV function with apical akinesis	A patent LAD stent and no obstructive disease	Positive	NR	NR
Boscolo‐Berto et al. (2021)^74^	Negative T waves over the inferior/anterior leads	Apical ballooning. mid‐ventricular to apical ballooning (asterisk) with preserved basal contraction (blues arrows) and a moderately impaired left ventricular ejection fraction of 40%	No coronary artery disease	Positive	Positive	Negative
Fearon et al. (2021)^75^	ST wave changes concerning for infero lateral ischemia and new poor anterior R wave progression	Mid‐ventricular ballooning of the LV, EF 20% with a Grade I diastolic dysfunction, mild mitral regurgitation, and severe right ventricular dysfunction associated with functional severe tricuspid regurgitation	No significant coronary artery disease	Positive	Positive	NR
Crane et al. (2021)^76^	Sinus tachycardia with first degree and right bundle branch block without acute or dynamic ischemic changes	New moderately severe segmental systolic dysfunction with an estimated ejection fraction of 37%–39% with hyperdynamic base, akinesis of the mid‐distal left ventricular segments and severe hypokinesis of the apical cap with apical ballooning	Patent grafts, no new flow limiting coronary disease and left ventriculography consistent with transthoracic echocardiogram finding with apical ballooning and reduced cardiac function in the antero‐apical regions	Positive	NR	NR
Stewart et al. (2021)^77^	Anterior T wave inversion with a corrected QT interval of 480ms, which evolved over 48 h	Hypokinesia of the mid‐cavity anteroseptum and the apical septum with overall mildly impaired left ventricular systolic contraction. No significant valvular heart disease, normal dimensions of the atria and ventricles and good right ventricular systolic contraction were noted	Left ventricular hypokinesia of the mid‐cavity anterior wall with no significant coronary artery disease present and left dominant coronary arteries	Positive	NR	NR
Tedeschi et al. (2022)^78^	Sinus rhythm with normal atrioventricular conduction, deep and symmetric T‐wave inversion in all leads except for aVL and aVF, and prolongation of corrected QT (QTc) >600 ms	Moderate depression of left ventricular contraction (LVEF 38%) in the presence of hypokinesia of apical and mid‐distal walls consistent with the apical ballooning syndrome	Non‐obstructive coronary artery disease	Positive	NR	NR
Toida et al. (2022)^79^	Atrial fibrillation with a normal axis, negative T‐waves in I, aVL, and V3‐6, and a prolonged QTc interval of 495 ms	Akinesia of the apical segments of the LV with apical ballooning and sparing of the base of each wall as well as a reduced ejection fraction of 48%. Systolic anterior motion of the mitral valve (MV) and LV outfow tract obstruction with basal hyperkinesia were detected in addition to mild‐moderate mitral regurgitation. Peak fow velocity and the mean pressure gradient of the LV outfow tract were 4.2 m/s and 71 mmHg, respectively	Not performed (coronary computed tomography showed no significant stenosis of the coronary arteries and extensive akinesis in the apical portion and hyperkinesia in the basal portion of LV with apical ballooning)	Positive	NR	Negative
Yamaura et al. (2022)^80^	ST‐segment depression on the V4–V6 leads	Akinesis at the basal portion of the left ventricle (LV) and hypercontraction at the apex	Not performed (coronary computed tomography angiography showed no significant stenosis in epicardial coronary arteries or aortic dissection. Coronary computed tomography angiography depicted akinesis at the basal portion of the LV, as visualized using transthoracic Doppler echo‐cardiography)	Positive	NR	Positive

Abbreviations: BNP, brain natriuretic peptide; NR, not reported; RVR, rapid ventricular rate; TTE, transthoracic echocardiogram.

Neurologic or psychiatric disorders (i.e., multiple sclerosis, cerebrovascular accident, dementia, schizophrenia, anxiety, depression, chronic pain syndrome, obstructive sleep apnea, and Bickerstaff brainstem encephalitis) were reported in 16/82 cases (19.5%) (Table 4). Among the case reports, the quality scores ranged from 5 to 8, in which 41 articles had the complete scores and providing the demographic characteristics and diagnostic tests were the items with the highest quality (Supporting Information: Table S2). Among the case series, the scores ranged from 6 to 10 with an average of 7.4. Validity of measuring the condition, consecutive, and complete inclusion of participants were those with the lowest quality in the included articles (Supporting Information: Table S3). The cohort study received the highest score for all of the items (Supporting Information: Table S4).

**Table 4:** Different physical and psychological etiologies associated with takotsubo in patients with COVID‐19

Study, Year	Study design	Transient left ventricular dysfunction in echocardiography	Emotional, physical, or combined trigger	Psychiatric/Neurologic disorders	New ECG abnormalities	Elevated cardiac biomarkers	Menopause
Takotsubo after COVID‐19							
Nguyen et al. (2020)^19^	Case report	Positive	Positive COVID‐19	Negative	Positive	Positive	Positive
Panchal et al. (2020)^20^	Case report	Positive	Positive COVID‐19	Negative	Positive	Negative	N/A
Kariyanna et al. (2020)^21^	Case report	Positive	Positive COVID‐19	Negative	Positive	Positive	Positive
Alizadehasl et al. (2022)^22^	Case report	Positive	Positive COVID‐19	Negative	Positive	Positive	N/A
Fujisaki et al. (2021)^23^	Case report	Positive	Positive COVID‐19	Negative	Positive	Positive	N/A
Demertzis et al. (2020)^24^	Case series	Positive	Positive COVID‐19	Positive	Negative	Positive	Positive
		Positive	Positive COVID‐19	Negative	Negative	Positive	Positive
Torabi et al. (2021)^25^	Case report	Positive	Positive COVID‐19	Negative	Negative	Positive	NR
Ortuno et al. (2021)^26^	Case report	Positive	Positive COVID‐19	Negative	Positive	Positive	N/A
Hegde et al. (2020)^27^	Case series	Positive	Positive COVID‐19	Negative	Positive	Positive	Positive
		Positive	Positive COVID‐19	Positive	Positive	Positive	N/A
		Positive	Positive COVID‐19	Negative	Positive	Negative	Positive
		Positive	Positive COVID‐19	Positive	Positive	Negative	Positive
		Positive	Positive COVID‐19	Positive	Positive	Positive	N/A
		Positive	Positive COVID‐19	Negative	Positive	Positive	N/A
		Positive	Positive COVID‐19	Positive	Positive	Positive	N/A
Hoepler et al. (2021)^28^	Case series	Positive	Positive COVID‐19	Negative	Positive	Positive	Positive
		Positive	Positive COVID‐19	Positive	Positive	Positive	Positive
		Positive	Positive COVID‐19	Positive	Positive	Positive	Positive
Alshamam et al. (2021)^4^	Case report	Positive	Positive COVID‐19	Negative	Positive	Positive	Positive
Bernardi et al. (2020)^29^	Case report	Positive	Positive COVID‐19	Negative	Positive	Positive	N/A
Sattar et al. (2020)^30^	Case report	Positive	Positive COVID‐19	Negative	Positive	Positive	NR
Tsao et al. (2020)^31^	Case report	Positive	Positive COVID‐19	Negative	Positive	Positive	NR
Gomez et al. (2020)^32^	Case report	Positive	Positive COVID‐19	Negative	Positive	Positive	NR
Belli et al. (2021)^18^	Case report	Positive	Positive COVID‐19	Negative	Positive	Positive	NR
Titi et al. (2020)^33^	Case report	Positive	Positive COVID‐19	Negative	Positive	NR	N/A
Faqihi et al. (2020)^34^	Case report	Positive	Positive COVID‐19	Negative	Positive	Positive	N/A
Solano‐López et al. (2020)^35^	Case report	Positive	Positive COVID‐19	Negative	Positive	Positive	N/A
Pasqualetto et al. (2020)^36^	Case series	Positive	Positive COVID‐19	Negative	Positive	Positive	N/A
		Positive	Positive COVID‐19	Negative	Positive	Positive	Positive
		Positive	Positive COVID‐19	Negative	Positive	Positive	N/A
Koh et al. (2021)^37^	Case report	Positive	Positive COVID‐19	Negative	Positive	Positive	N/A
Dave et al. (2020)^38^	Case report	Positive	Positive COVID‐19	Negative	Positive	Positive	Positive
Van Osch et al. (2020)^39^	Case report	Positive	Positive COVID‐19	Negative	Positive	Positive	Positive
Bhattacharyya et al. (2020)^40^	Case report	Positive	Positive COVID‐19	Negative	Positive	Positive	Negative
Taza et al. (2020)^41^	Case report	Positive	Positive COVID‐19	Positive	Positive	Negative	N/A
Bottiroli et al. (2020)^42^	Case report	Positive	Positive COVID‐19	Negative	Positive	Positive	Positive
Roca et al. (2020)^43^	Case report	Positive	Positive COVID‐19	Negative	Positive	Positive	Positive
Oyarzabal et al. (2020)^44^	Case report	Positive	Positive COVID‐19	Negative	Positive	NR	N/A
Minhas et al. (2020)^45^	Case report	Positive	Positive COVID‐19	Negative	Positive	Positive	Positive
Kong et al. (2021)^46^	Case series	Positive	Positive COVID‐19	Positive	Positive	Positive	N/A
		Positive	Positive COVID‐19	Positive	Positive	Positive	Positive
Park et al. (2020)^47^	Case series	Positive	Positive COVID‐19	Negative	Positive	Positive	Positive
		Positive	Positive COVID‐19	Negative	NR	Positive	Positive
Meyer et al. (2020)^48^	Case report	Positive	Positive COVID‐19	Negative	Positive	Positive	Positive
Eftekharzadeh et al. (2022)^49^	Case report	Positive	Positive COVID‐19	Positive	Positive	Positive	Positive
Frynas‐Jończyk et al. (2022)^50^	Case report	Positive	Positive COVID‐19	Negative	Positive	Positive	Positive
Fujiyoshi et al. (2022)^51^	Case report	Positive	Positive COVID‐19	Positive	Positive	Positive	Positive
Kimura et al. (2021)^52^	Case report	Positive	Positive COVID‐19	Positive	Positive	Positive	Positive
Mishra et al. (2021)^53^	Case report	Positive	Positive COVID‐19	Negative	Positive	NR	N/A
Namburu et al. (2021)^54^	Case report	Positive	Positive COVID‐19	Negative	Positive	Positive	N/A
Rivera et al. (2020)^55^	Case report	NR (but positive for angiography)	Positive COVID‐19	Negative	Positive	NR	Positive
Wildermann et al. (2022)^56^	Case report	NR (but positive for angiography)	Positive COVID‐19	Positive	Positive	Positive	Negative
Bapat et al. (2020)^57^	Case report	Positive	Positive COVID‐19	Negative	Positive	Positive	Positive
Chao et al. (2020)^58^	Case report	Positive	Positive COVID‐19	Negative	Positive	Positive	Positive
Dabbagh et al. (2020)^59^	Case report	Positive	Positive COVID‐19	Negative	Positive	Positive	Positive
Manzur‐Sandoval et al. (2021)^60^	Case report	Positive	Positive COVID‐19	Negative	Positive	Positive	NR
Sang et al. (2020)^61^	Case report	Positive	Positive COVID‐19	Negative	Positive	Positive	Positive
Tutor et al. (2021)^62^	Case series	Positive	Positive COVID‐19	Negative	Positive	Positive	N/A
		Positive	Positive COVID‐19	Negative	Positive	Positive	N/A
Takotsubo after social stress							
Habedank et al. (2020)^63^	Case report	Positive	Social stress/social isolation	Positive	Positive	Positive	Positive
Giannitsi et al. (2020)^64^	Case report	Positive	Death anxiety	Negative	Positive	Positive	Positive
Parker et al. (2020)^65^	Case report	Positive	phone consult which the patient was informed that the lung biopsy demonstrated recurrence of lung adenocarcinoma	Negative	Positive	Positive	Positive
Uhe et al. (2020)^66^	Case report	Positive	Death anxiety	Negative	Positive	Positive	Positive
Chadha et al. (2020)^67^	Case report	Positive	Social stress & death anxiety	Negative	Positive	Positive	Positive
Rivers et al. (2020)^68^	Case report	Positive	Social stress/social isolation	Negative	Positive	Positive	Positive
Koutroumpakis et al. (2020)^69^	Case report	Positive	Death anxiety	Negative	Positive	Positive	Positive
Jabri et al. (2020)^5^	Cohort	NR	Social stress/social isolation	NR	NR	Positive	NR
Dolci et al. (2020)^70^	Case report	Positive	Social stress/social isolation	Negative	Positive	Positive	Positive
Moady et al. (2021)^71^	Case series	Positive	Social stress/social isolation	Negative	Positive	Positive	Positive
		Positive	Social stress & death anxiety	Negative	Positive	Positive	Positive
Kir et al. (2021)^9^	Case series	Positive	Social stress/social isolation	Negative	Positive	Positive	Positive
		Positive	Death anxiety	Negative	Positive	NR	Positive
Mohammed et al. (2020)^72^	Case report	Positive	Increased workload	Negative	Positive	Positive	Positive
Ben Ammar et al. (2021)^73^	Case report	Positive	Social stress/social isolation	Positive	Positive	Positive	N/A
Takotsubo after COVID‐19 vaccination							
Vidula et al. (2021)^11^	Case series	Positive	Receiving second dose of the BNT162b2 (Pfizer‐BioNTech) vaccine	Negative	Positive	Positive	Positive
Tedeschi et al. (2022)^78^	Case report	Positive	Receiving first dose of the BNT162b2 (Pfizer–BioNTech) vaccine	Negative	Positive	Positive	Positive
Toida et al. (2022)^79^	Case report	Positive	Receiving first dose of the BNT162b2 (Pfizer‐BioNTech) vaccine	Negative	Positive	Positive	Positive
Yamaura et al. (2022)^80^	Case report	Positive	Receiving second dose of the BNT162b2 (Pfizer‐BioNTech) vaccine	Negative	Positive	Positive	Negative
Boscolo Berto et al. (2021)^74^	Case report	Positive	Receiving the first of two mRNA‐1273 (Moderna) COVID‐19 vaccinations	Negative	Positive	Positive	Positive
Fearon et al. (2021)^75^	Case report	Positive	Receiving first dose of the mRNA‐1273 (Moderna) vaccine	Negative	Positive	Positive	Positive
Crane et al. (2021)^76^	Case report	Positive	Receiving first dose of the ChadOX1 nCOV‐19 (AstraZeneca) vaccine	Negative	Negative	Positive	N/A
Stewart et al. (2021)^77^	Case report	Positive	Receiving second dose of the ChadOX1 nCOV‐19 (AstraZeneca) vaccine	Negative	Positive	Positive	Positive

Abbreviations: COVID‐19, coronavirus disease, 2019; ECG, electrocardiogram; N/A, not/applicable; NR, not reported.

## 4 DISCUSSION

The findings of the present study showed that the most common comorbidities and clinical presentation among those with TTS and COVID‐19 were hypertension and dyspnea, respectively. Moreover, the most common ECG findings of patients with COVID‐19 who developed TTS were ST elevation and T inversion. Elevated troponin, followed by BNP were the cardiac biomarkers which had the highest frequency among the patients. TTS leads to complications in which cardiogenic shock is the most common one. Comparing the patients with TTS after COVID‐19 infection and those with conventional TTS associated with emotional triggers shows that the most common presentation in the former group is dyspnea, some of them need mechanical ventilation, and the most common complication is cardiogenic shock, while in the latter group they mostly presented with chest pain, there is no need for mechanical ventilation, and the frequency of complications is lower.

In accordance with our study which found that most cases of TTS were developed in patients with COVID‐19 above 60 years of age, a systematic review conducted by Singh et al.^12^ on 12 case reports of TTS in patients with COVID‐19 showed a mean age of 70.8 years and 66.6% had age of above 60 years old. Moreover, the TTS was more common among women with COVID‐19 than males (66.6% vs. 33.4%),^16^ as it was also revealed in our study (69.5% vs. 30.5%). The same study also showed that hypertension (66.7%), followed by diabetes (41.6%), and dyslipidemia (16.6%) were the most common comorbidities.^12^ Similarly, we found that hypertension with a frequency of 65% was the most common comorbidity in these patients. The study also showed a mean time interval of 8.3 days from the first clinical presentation to admission which was almost in accordance with our study that was 7.2 days.^12^ In addition, Haussner et al.^81^ showed that the mean age of patients with COVID‐19 who developed TTS were higher than those with other COVID‐19‐assocaited cardiomyopathies (58.9 vs. 53.8 years).^81^

A systematic review on 99 studies, including 108 patients, showed that dyspnea (70.5%), chest pain (24.8%), and syncope (2.9%) had the highest frequency in patients with coexisting respiratory disease and TTS.^82^ In patients receiving mRNA COVID‐19 vaccines which lead to cardiac consequences, the most common signs/symptoms were chest pain (96.1%) and fever (38.2%).^83^ TTS can also clinically be presented with chest pain and dyspnea.^84^ Although most of the studies reported almost similar frequency in clinical presentations, minor differences could be as a result of differences in mean age, sex, and underlying diseases of the patients included in the different studies.

The ST‐T abnormalities, in particular ST‐segment elevation, were the most common ECG finding in patients with COVID‐19.^85^ Moreover, TTS could lead to occurrence of ST‐segment abnormalities.^86^ Diffuse ST elevation (43.8%) followed by PR depression (9.5%) were the most common ECG findings of those patients with cardiac complications after mRNA COVID‐19 vaccine administration.^83^ Among patients with COVID‐19 and TTS, only three‐fourth of patients had abnormal ECG in which ST elevation (50%), T inversion (50%), and prolonged QT‐interval (50%) were the most common findings.^12^ Similarly, the included studies in the current systematic review reported ST‐segment elevation, T‐wave inversion, and QT interval prolongation in 36.9%, 44.0%, and 16.6% of patients, respectively. The differences between the studies could be as a result of variations in the frequency of comorbidities among the included participants in the study which affect the ECG abnormalities.

In echocardiography, the mean LVEF was 36.4% and the GGO feature was reported in CXR of 31.9% of participants with COVID‐19 who developed TTS. In this regard, Singh et al.^12^ reported a mean 40.6% of LVEF of included 12 patients, and bilateral opacities (72.7%) and GGO (54.5%) were the most frequent findings in CXR.^12^ The minor differences between the studies could be as a result of higher number of included cases in our study than the previously conducted one in 2020.^12^ A preprint systematic review on eight case reports which aimed to evaluate the features of TTS following COVID‐19 vaccine administration showed that all cases had abnormal ECG and an elevated troponin test, while all of them had LVEFs of above 50%.^87^ In a study on 1216 patients with COVID‐19 who underwent echocardiography, evidence in favor of TTS was the least frequent cardiac complications that only found in solely 2% of them.^88^ The typical features of TTS in echocardiography are basal hypercontractility and apical ballooning.^89^ In this regard, we found the atypical ballooning in 38.8% of participants. Moreover, another systematic review on patients with COVID‐19 and TTS revealed atypical ballooning and basal segment hypo‐ or akinesia in 58.3% and 33.3% of participants, respectively.^12^

Elevated troponin, BNP, and CK were found to be increased in 96.1%, 95.0%, and 48.2% of included participants, respectively. Moreover, cardiac biomarkers, including NT‐proBNP, CK, troponin, and CK‐MB were increased in 28%, 18%, 17%, and 12% of patients with COVID‐19, respectively.^90^ Among those presented with cardiac complications following receiving COVID‐19 vaccines, CK‐MB, troponin, and NT‐proBNP were increased in 100%, 99.5%, and 78.3% of participants, respectively.^83^ The previously mentioned systematic review on patients with TTS and COVID‐19 also reported elevated troponin in 91.6% of participants.^12^ Therefore, troponin might be useful to be measured in patients with clinical presentations compatible with TTS in patients with positive COVID‐19. However, it needs to be confirmed with further diagnostic measures like echocardiography.

The TTS in patients with COVID‐19 could be life‐threatening as we found a mortality rate of 36.3%. Regarding the outcomes of patients with TTS and COVID‐19, the study by Singh et al.^12^ also found that 11 out of 12 patients developed at least one of the following complications: cardiac tamponade, heart failure, myocarditis, hypertensive crisis, septic shock, and cardiogenic shock. Nevertheless, the rate of recovery was almost high (90.9%).^12^ These differences could be due to including a higher number of cases in our systematic review and also differences in the number of patients with different past medical history and the medications used by them. Additionally, a systematic review of 123 patients with TTS during the COVID‐19 pandemic showed an in‐hospital morality rate of 23.3% which was significantly different by sex (38.7% in males and 13.9% in females; *p* = 0.03).^91^

There are some limitations in the present study which should be considered in the interpretation of the results. Firstly, because of the limited original papers on the association between TTS and COVID‐19, we only included case reports and case series. It could lead to bias since these types of papers are not indicative. Additionally, we did not exclude those studies with a past medical or family history of SARS‐CoV‐2 infection or cardiac diseases, so the previous infection with SARS‐CoV‐2 or a family history of cardiac diseases could susceptible the individuals to development of TTS and lead to bias in interpretation of the findings. Secondly, there is a probability of missing some relevant articles, although we used a comprehensive search strategy for electronic databases and gray literature search, as well as backward and forward citation searching. Thirdly, the relevant data for some items like CXR, ECG findings, or cardiac biomarkers, and complications were not reported by some studies. Fourthly, most of the included studies evaluated the TTS in patients with COVID‐19, while the TTS among those receiving COVID‐19 vaccines has not been comprehensively evaluated in the present study. Fifthly, most of the included studies were conducted in the United States, so due to the racial effects on the outcomes of TTS,^92^ the findings cannot be generalized to other races/ethnicities. Sixthly, pathological feature of TTS in patients with COVID‐19 or their cardiac computed tomography scans or magnetic resonance imaging findings were not reported in this systematic review. In addition, the treatments or drugs which were used for treatment of the individuals were not reported in the present study.

## 5 CONCLUSIONS

The TTS in patients with COVID‐19 is almost rare, whereas it could lead to a great mortality and morbidity. If an individual with COVID‐19, especially an elderly woman presented with dyspnea, ECG abnormality (e.g., ST elevation and T inversion) in addition to a rise in BNP and troponin and a decrease in LVEF, TTS should be considered as a differential diagnosis. Further observational studies and meta‐analyses on these studies are required to determine the association between COVID‐19 and TTS. Furthermore, the effects of COVID‐19 or history of previous SARS‐CoV‐2 infection on TTS development should be evaluated in future research. Future research should also focus on mechanisms related to TTS caused by COVID‐19 and if DNA samples can be extracted from these cases, it could lead to a new breakthrough in mechanistic research on COVID‐19‐induced TTS.

## AUTHOR CONTRIBUTIONS

**Hoomaan Ghasemi**: Conceptualization; methodology; project administration; visualization; writing – original draft; writing – review & editing. **Sina Kazemian**: Conceptualization; methodology; project administration; supervision; visualization; writing – original draft; writing – review & editing. **Seyed Aria Nejadghaderi**: Methodology; visualization; writing – original draft; writing – review & editing. **Mahan Shafie**: Conceptualization; investigation; methodology; project administration; supervision; validation; visualization; writing – original draft; writing – review & editing.

## CONFLICT OF INTEREST

The authors declare no conflict of interest.

## ETHICS STATEMENT

Since the ethical approval and the Institutional Review Board (IRB) were reported for each of the included studies, no additional ethical or IRB approvals were required for this systematic review.

## TRANSPARENCY STATEMENT

The lead author Mahan Shafie affirms that this manuscript is an honest, accurate, and transparent account of the study being reported; that no important aspects of the study have been omitted; and that any discrepancies from the study as planned (and, if relevant, registered) have been explained.

## Supporting information

Supporting information.

## Data Availability

The data that support the findings of this study are available from the corresponding author upon reasonable request.

## References

[1] Brenner ZR , Powers J . Takotsubo cardiomyopathy. Heart Lung. 2008;37(1):1‐7. 18206521 10.1016/j.hrtlng.2006.12.003

[2] Komamura K , Fukui M , Iwasaku T , Hirotani S , Masuyama T. Takotsubo cardiomyopathy: pathophysiology, diagnosis and treatment. World J Cardiol. 2014;6(7):602‐609. 25068020 10.4330/wjc.v6.i7.602 PMC4110608

[3] Gianni M , Dentali F , Grandi AM , Sumner G , Hiralal R , Lonn E . Apical ballooning syndrome or takotsubo cardiomyopathy: a systematic review. Eur Heart J. 2006;27(13):1523‐1529. 16720686 10.1093/eurheartj/ehl032

[4] Alshamam MS , Nso N , Idrees Z , Nassar M , Munira MS . Coronavirus disease 2019 (COVID‐19)‐induced takotsubo cardiomyopathy prognosis in geriatric setting. Cureus. 2021;13(7):e16211. 34367812 10.7759/cureus.16211 PMC8341288

[5] Jabri A , Kalra A , Kumar A , et al. Incidence of stress cardiomyopathy during the coronavirus disease 2019 pandemic. JAMA Network Open. 2020;3(7):e2014780. 32644140 10.1001/jamanetworkopen.2020.14780 PMC7348683

[6] Desai HD , Sharma K , Jadeja DM , Desai HM , Moliya P . COVID‐19 pandemic induced stress cardiomyopathy: a literature review. IJC Heart Vasc. 2020;31:100628. 10.1016/j.ijcha.2020.100628 PMC7476902 32923579

[7] Sattar Y , Ullah W , Rauf H , et al. COVID‐19 cardiovascular epidemiology, cellular pathogenesis, clinical manifestations and management. IJC Heart Vasc. 2020;29:100589. 10.1016/j.ijcha.2020.100589 PMC7359794 32724831

[8] Turshudzhyan A . Severe acute respiratory syndrome coronavirus 2 (SARS‐CoV‐2)‐induced cardiovascular syndrome: etiology, outcomes, and management. Cureus. 2020;12(6):e8543. 32670680 10.7759/cureus.8543 PMC7357341

[9] Kir D , Beer N , De Marchena EJ . Takotsubo cardiomyopathy caused by emotional stressors in the coronavirus disease 2019 (COVID‐19) pandemic era. J Card Surg. 2021;36(2):764‐769. 33336409 10.1111/jocs.15251

[10] Mahan S , Mahsa M , Hamed H , Mahnaz A . Medical sciences' students responses during the late phase of the COVID‐19 pandemic in Iran: a comprehensive investigation of the risk perception and information exposure. Acta Med Iranica. 2021;59(12):704‐712.

[11] Vidula MK , Ambrose M , Glassberg H , et al. Myocarditis and other cardiovascular complications of the mRNA‐based COVID‐19 vaccines. Cureus. 2021;13(6):e15576. 34277198 10.7759/cureus.15576 PMC8270057

[12] Singh S , Desai R , Gandhi Z , et al. Takotsubo syndrome in patients with COVID‐19: a systematic review of published cases. SN Compr Clin Med. 2020;2(11):2102‐2108. 33043251 10.1007/s42399-020-00557-w PMC7538054

[13] Page MJ , McKenzie JE , Bossuyt PM , et al. The PRISMA 2020 statement: an updated guideline for reporting systematic reviews. Syst Rev. 2021;10(1):89. 33781348 10.1186/s13643-021-01626-4 PMC8008539

[14] Madhavan M , Prasad A . Proposed Mayo Clinic criteria for the diagnosis of Tako‐Tsubo cardiomyopathy and long‐term prognosis. Herz. 2010;35(4):240‐244. 20582391 10.1007/s00059-010-3339-x

[15] Institute JB . *Checklist for case reports*. 2020. https://jbi.global/critical-appraisal-tools

[16] Institute JB . *Checklist for case series*. 2020. https://jbi.global/critical-appraisal-tools

[17] Institute JB . *Checklist for cohort studies*. 2020. https://jbi.global/critical-appraisal-tools

[18] Belli O , Ardissino M , Bottiroli M , et al. Emergency cardiac imaging for coronavirus disease 2019 (COVID‐19) in practice: a case of takotsubo stress cardiomyopathy. Cardiovasc Ultrasound. 2021;19(1):31. 34429107 10.1186/s12947-021-00251-4 PMC8383239

[19] Nguyen D , Nguyen T , De Bels D , Castro Rodriguez J . A case of takotsubo cardiomyopathy with COVID 19. Eur Heart J Cardiovasc Imaging. 2020;21(9):1052. 32395765 10.1093/ehjci/jeaa152 PMC7239208

[20] Panchal A , Kyvernitakis A , Biederman R . An interesting case of COVID‐19 induced reversed takotsubo cardiomyopathy and insight on cardiac biomarkers. Cureus. 2020;12(11):e11296. 33282573 10.7759/cureus.11296 PMC7710340

[21] Kariyanna PT , Chandrakumar HP , Jayarangaiah A , et al. Apical takotsubo cardiomyopathy in a COVID‐19 patient presenting with stroke: a case report and pathophysiologic insights. Am J Med Case Rep. 2020;8(10):350‐357.32704530

[22] Alizadehasl A , Soleimani A , Peighambari MM , Mostafavi A . Biventricular apical ballooning in patient with COVID‐19. J Echocardiogr. 2022;20:243‐244. 33982248 10.1007/s12574-021-00530-z PMC8115864

[23] Fujisaki T , Kassim F , Kassim G , Bandyopadhyay D , Singh V , Kim B . Biventricular takotsubo syndrome with COVID‐19 in an Asian male. J Cardiol Cases. 2021;24(1):6‐9. 33262862 10.1016/j.jccase.2020.11.017 PMC7690273

[24] Demertzis ZD , Dagher C , Malette KM , et al. Cardiac sequelae of novel coronavirus disease 2019 (COVID‐19): a clinical case series. Eur Heart J. 2020;4(FI1):1‐6. 10.1093/ehjcr/ytaa179 PMC7314080 33089042

[25] Torabi AJ , Villegas‐Galaviz J , Guglin M , Frick K , Rao R . Cardiogenic shock following cardiac tamponade and takotsubo in COVID‐19. Future Cardiol. 2021;17(4):631‐635. 33078963 10.2217/fca-2020-0115 PMC7574646

[26] Ortuno S , Jozwiak M , Mira J‐P , Nguyen LS . Case report: takotsubo syndrome associated with novel coronavirus disease 2019. Front Cardiovasc Med. 2021;8:614562. 33693034 10.3389/fcvm.2021.614562 PMC7937625

[27] Hegde S , Khan R , Zordok M , Maysky M . Characteristics and outcome of patients with COVID‐19 complicated by takotsubo cardiomyopathy: case series with literature review. Open Heart. 2020;7(2):e001360. 33020258 10.1136/openhrt-2020-001360 PMC7536639

[28] Hoepler W , Traugott MT , Christ G , et al. Clinical and angiographic features in three COVID‐19 patients with takotsubo cardiomyopathy. case report. SN Compr Clin Med. 2021;3(1):263‐268. 33426474 10.1007/s42399-020-00683-5 PMC7786154

[29] Bernardi N , Calvi E , Cimino G , et al. COVID‐19 pneumonia, takotsubo syndrome, and left ventricle thrombi. JACC Case Rep. 2020;2(9):1359‐1364. 32835280 10.1016/j.jaccas.2020.06.008 PMC7290218

[30] Sattar Y , Connerney M , Ullah W , et al. COVID‐19 presenting as takotsubo cardiomyopathy complicated with atrial fibrillation. IJC Heart Vasc. 2020;29:100580. 10.1016/j.ijcha.2020.100580 PMC7348613 32685662

[31] Tsao CW , Strom JB , Chang JD , Manning WJ . COVID‐19‐associated stress (takotsubo) cardiomyopathy. Circ Cardiovasc Imaging. 2020;13(7):e011222. 32673494 10.1161/CIRCIMAGING.120.011222 PMC7398589

[32] Gomez JMD , Nair G , Nanavaty P , Rao A , Marinescu K , Suboc T . COVID‐19‐associated takotsubo cardiomyopathy. BMJ Case Rep. 2020;13(12):e236811. 10.1136/bcr-2020-236811 PMC7735089 33310830

[33] Titi L , Magnanimi E , Mancone M , et al. Fatal takotsubo syndrome in critical COVID‐19 related pneumonia. Cardiovasc Pathol. 2021;51:107314. 33259936 10.1016/j.carpath.2020.107314 PMC7699026

[34] Faqihi F , Alharthy A , Alshaya R , et al. Reverse takotsubo cardiomyopathy in fulminant COVID‐19 associated with cytokine release syndrome and resolution following therapeutic plasma exchange: a case‐report. BMC Cardiovasc Disord. 2020;20(1):389. 32842957 10.1186/s12872-020-01665-0 PMC7447602

[35] Solano‐López J , Sánchez‐Recalde A , Zamorano JL . SARS‐CoV‐2, a novel virus with an unusual cardiac feature: inverted takotsubo syndrome. Eur Heart J. 2020;41(32):3106. 32391885 10.1093/eurheartj/ehaa390 PMC7239192

[36] Pasqualetto MC , Secco E , Nizzetto M , et al. Stress cardiomyopathy in COVID‐19 disease. Eur J Case Rep Intern Med. 2020;7(6):001718. 32523926 10.12890/2020_001718 PMC7279910

[37] Koh MCY , Li TYW , Ong JSY , Somani J , Ambhore AA . Stress cardiomyopathy with transient biventricular dysfunction following recent COVID‐19 infection. Acta Cardiol Sin. 2021;37(2):204‐207. 33716463 10.6515/ACS.202103_37(2).20201221A PMC7953119

[38] Dave S , Thibodeau JT , Styrvoky K , Bhatt SH . Takotsubo cardiomyopathy in a coronavirus disease‐2019‐positive patient: a case report. A&A Pract. 2020;14(11):e01304. 10.1213/XAA.0000000000001304 PMC8330628 32985848

[39] van Osch D , Asselbergs FW , Teske AJ . Takotsubo cardiomyopathy in COVID‐19: a case report. haemodynamic and therapeutic considerations. Eur Heart J Case Rep. 2020;4(Fi1):1‐6. 10.1093/ehjcr/ytaa271 PMC7528942 33437922

[40] Bhattacharyya PJ , Attri PK , Farooqui W . Takotsubo cardiomyopathy in early term pregnancy: a rare cardiac complication of SARS‐CoV‐2 infection. BMJ Case Rep. 2020;13(9):e239104. 10.1136/bcr-2020-239104 PMC7523204 32988978

[41] Taza F , Zulty M , Kanwal A , Grove D . Takotsubo cardiomyopathy triggered by SARS‐CoV‐2 infection in a critically ill patient. BMJ Case Rep. 2020;13(6):e236561. 10.1136/bcr-2020-236561 PMC7298682 32540884

[42] Bottiroli M , De Caria D , Belli O , et al. Takotsubo syndrome as a complication in a critically ill COVID‐19 patient. ESC Heart Fail. 2020;7(6):4297‐4300. 32886428 10.1002/ehf2.12912 PMC7754970

[43] Roca E , Lombardi C , Campana M , et al. Takotsubo syndrome associated with COVID‐19. Eur J Case Rep Intern Med. 2020;7(5):1. 10.12890/2020_001665 PMC7213829 32399453

[44] Oyarzabal L , Gómez‐Hospital JA , Comin‐Colet J . Síndrome de tako‐tsubo asociado con COVID‐19. Rev Esp Cardiol. 2020;73(10):846. 10.1016/j.recesp.2020.06.022 PMC7311895 32836659

[45] Minhas AS , Scheel P , Garibaldi B , et al. Takotsubo syndrome in the setting of COVID‐19. JACC Case Rep. 2020;2(9):1321‐1325. 32363351 10.1016/j.jaccas.2020.04.023 PMC7194596

[46] Kong N , Singh N , Mazzone S , Burkhart R , Anchan R , Blair J . Takotsubo syndrome presenting as cardiogenic shock in patients with COVID‐19: a case series and review of current literature. Cardiovasc Revasc Med. 2021;28s:50‐53.3351663810.1016/j.carrev.2021.01.017

[47] Park JH , Moon JY , Sohn KM , Kim YS . Two fatal cases of stress‐induced cardiomyopathy in COVID‐19 patients. J Cardiovas Imaging. 2020;28(4):300‐303. 10.4250/jcvi.2020.0125 PMC7572266 33086446

[48] Meyer P , Degrauwe S , Van Delden C , Ghadri JR , Templin C . Typical takotsubo syndrome triggered by SARS‐CoV‐2 infection. Eur Heart J. 2020;41(19):1860. 32285915 10.1093/eurheartj/ehaa306 PMC7184501

[49] Eftekharzadeh P , Patel A , Sokolova E , Rodas A , Ahmed S . Takotsubo cardiomyopathy: a COVID‐19 complication. Cureus. 2022;14(3):e22803. 35399473 10.7759/cureus.22803 PMC8980236

[50] Frynas‐Jończyk K , Wiek‐Rębowska E , Filipiak‐Strzecka D , Szymczyk E , Kasprzak JD . COVID‐tsubo: Takotsubo syndrome in patient hospitalized due to the SARS‐CoV‐2 infection. Pol Arch Intern Med. 2022;132:16255. 35522149 10.20452/pamw.16255

[51] Fujiyoshi K , Ako J , Ishida K , Ishida M , Minami Y , Inomata T . Tako‐tsubo‐like left ventricular dysfunction in a patient with COVID‐19 demonstrated by non‐invasive multi‐modality imaging. J Nucl Cardiol. 2022;29(2):863‐865. 33000408 10.1007/s12350-020-02367-y PMC7527244

[52] Kimura M , Hashiguchi S , Tanaka K , et al. Case report: takotsubo cardiomyopathy in Bickerstaff brainstem encephalitis triggered by COVID‐19. Front Neurol. 2021;12:822247. 35002947 10.3389/fneur.2021.822247 PMC8741194

[53] Mishra AK , Dai Q , Sahu KK , ElMeligy A . Atypical takotsubo cardiomyopathy in COVID‐19. Am J Med Sci. 2021;362(5):e41‐e42. 34446260 10.1016/j.amjms.2021.01.024 PMC7847281

[54] Namburu L , Bhogal S , Ramu VK . COVID‐19‐Induced takotsubo cardiomyopathy with concomitant pulmonary embolism. Cureus. 2021;13(10):e18693. 34659930 10.7759/cureus.18693 PMC8513724

[55] Rivera K , Fernández‐Rodríguez D , Zielonka M , Casanova‐Sandoval J . Diagnosis of takotsubo syndrome in the COVID‐19 era. Rev Port Cardiol. 2021;40(11):899‐901. 10.1016/j.repce.2021.10.027 PMC8628165 34857167

[56] Wildemann B , Jarius S , Lehmann LH , et al. COVID‐19‐related severe MS exacerbation with life‐threatening takotsubo cardiomyopathy in a previously stable patient and interference of MS therapy with long‐term immunity against SARS‐CoV‐2. J Neurol. 2022;269(3):1138‐1141. 34617145 10.1007/s00415-021-10779-0 PMC8494626

[57] Bapat A , Maan A , Heist EK . Stress‐induced cardiomyopathy secondary to COVID‐19. Case Rep Cardiol. 2020;2020:1‐6. 10.1155/2020/8842150 PMC7479482 32934848

[58] Chao CJ , DeValeria PA , Sen A , et al. Reversible cardiac dysfunction in severe COVID‐19 infection, mechanisms and case report. Echocardiography. 2020;37(9):1465‐1469. 32856328 10.1111/echo.14807 PMC7461431

[59] Dabbagh MF , Aurora L , D'Souza P , Weinmann AJ , Bhargava P , Basir MB . Cardiac tamponade secondary to COVID‐19. JACC Case Rep. 2020;2(9):1326‐1330. 32328588 10.1016/j.jaccas.2020.04.009 PMC7177077

[60] Manzur‐Sandoval D , Carmona‐Levario P , García‐Cruz E . Giant inverted T waves in a patient with COVID‐19 infection. Ann Emerg Med. 2021;77(2):264‐267. 33010955 10.1016/j.annemergmed.2020.07.037 PMC7402110

[61] Sang CJ 3rd , Heindl B , Von Mering G , et al. Stress‐Induced cardiomyopathy precipitated by COVID‐19 and influenza A coinfection. JACC Case Rep. 2020;2(9):1356‐1358. 32835279 10.1016/j.jaccas.2020.05.068 PMC7280126

[62] Tutor A , Unis G , Ruiz B , Bolaji OA , Bob‐Manuel T . Spectrum of suspected cardiomyopathy due to COVID‐19: A case series. Curr Probl Cardiol. 2021;46(10):100926. 34311983 10.1016/j.cpcardiol.2021.100926 PMC8254392

[63] Habedank D , Thieme R , Bublak A , Heinemann F , Spencker S , Atmowihardjo I . Ventricular fibrillation and takotsubo cardiomyopathy triggered by media panic on COVID‐19: a case report. Clin Case Rep. 2021;9(1):72‐76. 33362931 10.1002/ccr3.3423 PMC7753670

[64] Giannitsi S , Tsinivizov P , Poulimenos LE , et al. [Case report] Stress induced (takotsubo) cardiomyopathy triggered by the COVID‐19 pandemic. Exp Ther Med. 2020;20(3):2812‐2814. 32765776 10.3892/etm.2020.8968 PMC7401702

[65] Parker J , Niranjan S , Sriram KB . A case of broken heart syndrome via the telephone: socially distant outpatient clinics in the COVID‐19 pandemic. Intern Med J. 2020;50(11):1429‐1431. 32827338 10.1111/imj.14980 PMC7460991

[66] Uhe T , Hagendorff A , Wachter R , Laufs U . Collateral damage: fear from SARS‐CoV2‐infection causing takotsubo cardiomyopathy. Clin Res Cardiol. 2020;109(12):1588‐1594. 32661675 10.1007/s00392-020-01706-w PMC7356132

[67] Chadha S . COVID‐19 pandemic' anxiety‐induced takotsubo cardiomyopathy. QJM. 2020;113(7):488‐490. 32311043 10.1093/qjmed/hcaa135 PMC7188117

[68] Rivers J , Ihle JF . COVID‐19 social isolation‐induced takotsubo cardiomyopathy. Med J Aust. 2020;213(7):336‐e1. 32909275 10.5694/mja2.50770

[69] Koutroumpakis E , Taylor T , Damaraju S , Badruddin Mawji S . “Covidsubo”: stress‐induced cardiomyopathy by novel coronavirus disease 2019. Cardiology. 2020;145(12):779‐783. 32987390 10.1159/000511450 PMC7573897

[70] Dolci G , Prevedello F , Lobascio I , Mugnai G , Dalla Valle C , Bilato C . Mid‐ventricular takotsubo syndrome ‘lockdown'‐related during coronavirus disease 2019 outbreak: a case report. J Cardiovasc Med. 2021;22(5):414‐416. 10.2459/JCM.0000000000001054 32941322

[71] Moady G , Atar S . Quarantine‐induced stress cardiomyopathy (takotsubo syndrome) during the COVID‐19 pandemic. Isr Med Assoc J. 2021;23(3):149‐152. 33734626

[72] Mohammed M , Zakhour S , Devgun J , Lee J , Keimig T , Wang DD . Takotsubo cardiomyopathy in a healthcare worker during the COVID‐19 pandemic: caused by the virus or the demands of the many being placed on the few? Eur J Case Rep Intern Med. 2020;7(12):002088. 33313019 10.12890/2020_002088 PMC7727620

[73] Ben Ammar H , Bouguira E , Brahmi L , et al. Simultaneous occurrence of a takotsubo syndrome and paranoia delirium, related to Covid‐19 pandemic: a case report. Clin Case Rep. 2021;9(11):e05026. 34765206 10.1002/ccr3.5026 PMC8572332

[74] Boscolo Berto M , Spano G , Wagner B , et al. Takotsubo cardiomyopathy after mRNA COVID‐19 vaccination. Heart Lung Circ. 2021;30(12):e119‐e120. 34330629 10.1016/j.hlc.2021.06.521 PMC8279960

[75] Fearon C , Parwani P , Gow‐Lee B , Abramov D . Takotsubo syndrome after receiving the COVID‐19 vaccine. J Cardiol Cases. 2021;24(5):223‐226. 34539938 10.1016/j.jccase.2021.08.012 PMC8440167

[76] Crane P , Wong C , Mehta N , Barlis P . Takotsubo (stress) cardiomyopathy after ChAdOx1 nCoV‐19 vaccination. BMJ Case Rep. 2021;14(10):e246580. 10.1136/bcr-2021-246580 PMC8504353 34625447

[77] Stewart C , Gamble DT , Dawson D . Novel case of takotsubo cardiomyopathy following COVID‐19 vaccination. BMJ Case Rep. 2022;15(1):e247291. 10.1136/bcr-2021-247291 PMC8768865 35042734

[78] Tedeschi A , Camilli M , Ianni U , et al. Takotsubo syndrome after BNT162b2 mRNA Covid‐19 vaccine: emotional or causative relationship with vaccination? IJC Heart Vasc. 2022;40:101002. 10.1016/j.ijcha.2022.101002 PMC8934733 35340274

[79] Toida R , Uezono S , Komatsu H , et al. Takotsubo cardiomyopathy after vaccination for coronavirus disease 2019 in a patient on maintenance hemodialysis. CEN Case Rep. 2022;11(2):220‐224. 34731486 10.1007/s13730-021-00657-z PMC8564792

[80] Yamaura H , Ishikawa H , Otsuka K , Kasayuki N . Reverse takotsubo cardiomyopathy as a cause of acute chest pain in a young woman following COVID‐19 vaccination. Circ Cardiovasc Imaging. 2022;15(1):e013661. 34961327 10.1161/CIRCIMAGING.121.013661 PMC8772050

[81] Haussner W , DeRosa AP , Haussner D , et al. COVID‐19 associated myocarditis: a systematic review. Am J Emerg Med. 2022;51:150‐155. 34739868 10.1016/j.ajem.2021.10.001 PMC8531234

[82] Li P , Wang Y , Liang J , et al. Takotsubo syndrome and respiratory diseases: a systematic review. Eur Heart J Open. 2022;2(2):oeac009. 35919117 10.1093/ehjopen/oeac009 PMC9242042

[83] Fazlollahi A , Zahmatyar M , Noori M , et al. Cardiac complications following mRNA COVID‐19 vaccines: a systematic review of case reports and case series. Rev Med Virol. 2022;32:e2318. 34921468 10.1002/rmv.2318

[84] Amin HZ , Amin LZ , Pradipta A . Takotsubo cardiomyopathy: a brief review. J Med Life. 2020;13(1):3‐7. 32341693 10.25122/jml-2018-0067 PMC7175432

[85] Mehraeen E , Seyed Alinaghi SA , Nowroozi A , et al. A systematic review of ECG findings in patients with COVID‐19. Indian Heart J. 2020;72(6):500‐507. 33357637 10.1016/j.ihj.2020.11.007 PMC7661958

[86] Pelliccia F , Pasceri V , Patti G , et al. Long‐term prognosis and outcome predictors in takotsubo syndrome. JACC: Heart Fail. 2019;7(2):143‐154. 30611720 10.1016/j.jchf.2018.10.009

[87] Ahmed SK , Mohamed MG , Essa RA , et al. Global reports of takotsubo (stress) cardiomyopathy following COVID‐19 vaccination: a systematic review and meta‐analysis. J Cardiol Heart Vasc. 2022;43:101‐108. 10.1016/j.ijcha.2022.101108 PMC9381427 35992364

[88] Dweck MR , Bularga A , Hahn RT , et al. Global evaluation of echocardiography in patients with COVID‐19. Eur Heart J Cardiovasc Imaging. 2020;21(9):949‐958. 32556199 10.1093/ehjci/jeaa178 PMC7337658

[89] Moady G , Atar S . Takotsubo syndrome during the COVID‐19 pandemic: state‐of‐the‐art review. CJC Open. 2021;3(10):1249‐56. 34056570 10.1016/j.cjco.2021.05.011 PMC8149464

[90] Shafi AMA , Shaikh SA , Shirke MM , Iddawela S , Harky A . Cardiac manifestations in COVID‐19 patients—a systematic review. J Card Surg. 2020;35(8):1988‐2008. 32652713 10.1111/jocs.14808 PMC7404674

[91] Chang A , Wang YG , Jayanna MB , Wu X , Cadaret LM , Liu K . Mortality correlates in patients with takotsubo syndrome during the COVID‐19 pandemic. Mayo Clin Proc Innov Qual Outcomes. 2021;5(6):1050‐1055. 34604705 10.1016/j.mayocpiqo.2021.09.008 PMC8479536

[92] Zaghlol R , Dey AK , Desale S , Barac A . Racial differences in takotsubo cardiomyopathy outcomes in a large nationwide sample. ESC Heart Fail. 2020;7(3):1056‐1063. 32147963 10.1002/ehf2.12664 PMC7261569

