# Supplementary material for: Takotsubo syndrome and COVID‐19: A systematic review
Source: Health Sci Rep. 2022 Dec 2;6(1):e972. doi: 10.1002/hsr2.972 (PMC9718950; doi:10.1002/hsr2.972)
Supplement: Supplementary file 1 — Supporting information. [file HSR2-6-0-s001.docx]

**Table S1.** Search strategy of the three databases.

| **Database**  **(Search date: May 12, 2022)** | **Search** | **Query** | **Results (No.)** |
| --- | --- | --- | --- |
| PubMed | #1 | "Takotsubo Cardiomyopathy"[Mesh] OR “takotsubo cardiomyopathy”[All FIelds] OR “stress cardiomyopathy”[All Fields] OR “tako-tsubo cardiomyopathy”[All Fields] OR “tako tsubo cardiomyopathy”[All Fields] OR “broken heart syndrome*”[All Fields] OR “takotsubo syndrome*”[All Fields] OR “transient apical ballooning syndrome*”[All Fields] OR “apical ballooning syndrome*”[All Fields] OR “left ventricular apical ballooning syndrome*”[All Fields] OR “tako-tsubo syndrome*”[All Fields] OR “tako tsubo syndrome*”[All Fields] | 6,021 |
|  | #2 | "COVID-19"[Mesh] OR “COVID-19”[All Fields] OR “COVID 19”[All Fields] OR “COVID19”[All Fields] OR “COVID-19 Virus Disease*”[All Fields] OR “COVID 19 Virus Disease*”[All Fields] OR “COVID-19 Virus Infection*”[All Fields] OR “COVID 19 Virus Infection*”[All Fields] OR “2019-nCoV Infection*”[All Fields] OR “2019 nCoV Infection*”[All Fields] OR “Coronavirus Disease-19”[All Fields] OR “Coronavirus Disease 19”[All Fields] OR “2019 Novel Coronavirus Disease*”[All Fields] OR “2019 Novel Coronavirus Infection*”[All Fields] OR “2019-nCoV Disease*”[All Fields] OR “2019 nCoV Disease*”[All Fields] OR “Coronavirus Disease 2019”[All Fields] OR “SARS Coronavirus 2 Infection*”[All Fields] OR “SARS-CoV-2 Infection*”[All Fields] OR “SARS CoV 2 Infection*”[All Fields] OR “COVID-19 Pandemic*”[All Fields] OR “COVID 19 Pandemic*”[All Fields] OR "SARS-CoV-2"[Mesh] OR “Coronavirus Disease 2019 Virus*”[All Fields] OR “2019 Novel Coronavirus*”[All Fields] OR “Wuhan Seafood Market Pneumonia Virus*”[All Fields] OR “SARS-CoV-2 Virus*”[All Fields] OR “SARS CoV 2 Virus*”[All Fields] OR “2019-nCoV”[All Fields] OR “COVID-19 Virus*”[All Fields] OR “COVID 19 Virus*”[All Fields] OR “Wuhan Coronavirus*”[All Fields] OR “SARS Coronavirus 2”[All Fields] OR “Severe Acute Respiratory Syndrome Coronavirus 2”[All Fields] OR "COVID-19 Vaccines"[Mesh] OR “COVID 19 Vaccine*”[All Fields] OR “COVID-19 Virus Vaccine*”[All Fields] OR “COVID 19 Virus Vaccine*”[All Fields] OR “COVID-19 Virus Vaccine*”[All Fields] OR “COVID 19 Virus Vaccine*”[All Fields] OR “COVID19 Virus Vaccine*”[All Fields] OR “COVID19 Virus Vaccine*”[All Fields] OR “COVID19 Vaccine*”[All Fields] OR “COVID19 Vaccine*”[All Fields] OR “SARS-CoV-2 Vaccine*”[All Fields] OR “SARS CoV 2 Vaccine*”[All Fields] OR “SARS2 Vaccine*”[All Fields] OR “Coronavirus Disease 2019 Vaccine*”[All Fields] OR “Coronavirus Disease 2019 Virus Vaccine*”[All Fields] OR “Coronavirus Disease-19 Vaccine*”[All Fields] OR “Coronavirus Disease 19 Vaccine*”[All Fields] OR “COVID 19 Vaccine*”[All Fields] OR “2019-nCoV Vaccine*”[All Fields] OR “2019 nCoV Vaccine*”[All Fields] OR “2019 Novel Coronavirus Vaccine*”[All Fields] OR “2019-nCoV Vaccine*”[All Fields] OR “2019 nCoV Vaccine*”[All Fields] OR “COVID-19 Vaccine*”[All Fields] OR “SARS Coronavirus 2 Vaccine*”[All Fields] | 248,621 |
|  | #3 | #1 AND #2 | 180 |
| EMBASE | #1 | 'takotsubo cardiomyopathy'/mj/exp OR 'takotsubo cardiomyopathy' OR 'stress cardiomyopathy':ti,ab,kw OR 'tako-tsubo cardiomyopathy':ti,ab,kw OR 'tako tsubo cardiomyopathy':ti,ab,kw OR 'broken heart syndrome*':ti,ab,kw OR 'takotsubo syndrome*':ti,ab,kw OR 'transient apical ballooning syndrome*':ti,ab,kw OR 'apical ballooning syndrome*':ti,ab,kw OR 'left ventricular apical ballooning syndrome*':ti,ab,kw OR 'tako-tsubo syndrome*':ti,ab,kw OR 'tako tsubo syndrome*':ti,ab,kw | 10,726 |
|  | #2 | 'coronavirus disease 2019'/mj OR 'coronavirus disease 2019'/exp OR 'coronavirus disease 2019' OR 'sars-cov-2 vaccine'/mj OR 'sars-cov-2 vaccine'/exp OR 'sars-cov-2 vaccine' OR 'covid-19':ti,ab,kw OR 'covid 19':ti,ab,kw OR 'covid19':ti,ab,kw OR 'covid-19 virus disease*':ti,ab,kw OR 'covid 19 virus disease*':ti,ab,kw OR 'covid-19 virus infection*':ti,ab,kw OR 'covid 19 virus infection*':ti,ab,kw OR '2019-ncov infection*':ti,ab,kw OR '2019 ncov infection*':ti,ab,kw OR 'coronavirus disease-19':ti,ab,kw OR 'coronavirus disease 19':ti,ab,kw OR '2019 novel coronavirus disease*':ti,ab,kw OR '2019 novel coronavirus infection*':ti,ab,kw OR '2019-ncov disease*':ti,ab,kw OR '2019 ncov disease*':ti,ab,kw OR 'sars coronavirus 2 infection*':ti,ab,kw OR 'sars-cov-2 infection*':ti,ab,kw OR 'sars cov 2 infection*':ti,ab,kw OR 'covid-19 pandemic*':ti,ab,kw OR 'covid 19 pandemic*':ti,ab,kw OR 'coronavirus disease 2019 virus*':ti,ab,kw OR '2019 novel coronavirus*':ti,ab,kw OR 'wuhan seafood market pneumonia virus*':ti,ab,kw OR 'sars-cov-2 virus*':ti,ab,kw OR 'sars cov 2 virus*':ti,ab,kw OR '2019-ncov':ti,ab,kw OR 'covid-19 virus*':ti,ab,kw OR 'covid 19 virus*':ti,ab,kw OR 'wuhan coronavirus*':ti,ab,kw OR 'sars coronavirus 2':ti,ab,kw OR 'severe acute respiratory syndrome coronavirus 2':ti,ab,kw OR 'covid-19 virus vaccine*':ti,ab,kw OR 'covid 19 virus vaccine*':ti,ab,kw OR 'covid19 virus vaccine*':ti,ab,kw OR 'covid19 vaccine*':ti,ab,kw OR 'sars cov 2 vaccine*':ti,ab,kw OR 'sars2 vaccine*':ti,ab,kw OR 'coronavirus disease 2019 vaccine*':ti,ab,kw OR 'coronavirus disease 2019 virus vaccine*':ti,ab,kw OR 'coronavirus disease-19 vaccine*':ti,ab,kw OR 'coronavirus disease 19 vaccine*':ti,ab,kw OR 'covid 19 vaccine*':ti,ab,kw OR '2019 novel coronavirus vaccine*':ti,ab,kw OR '2019-ncov vaccine*':ti,ab,kw OR '2019 ncov vaccine*':ti,ab,kw OR 'covid-19 vaccine*':ti,ab,kw OR 'sars coronavirus 2 vaccine*':ti,ab,kw | 272,319 |
|  | #3 | ([article]/lim OR [article in press]/lim OR [data papers]/lim OR [editorial]/lim OR [erratum]/lim OR [letter]/lim OR [note]/lim OR [review]/lim OR [short survey]/lim) | 34,560,152 |
|  | #4 | #1 AND #2 AND #3 | 325 |
| Scopus | #1 | ( ALL ( covid-19 ) OR ALL ( covid19 ) OR ALL ( 2019-ncov AND infection ) OR ALL ( 2019 ncov AND infection ) OR ALL ( coronavirus AND disease-19 ) OR ALL ( coronavirus AND disease 19 ) OR ALL ( 2019 novel AND coronavirus AND disease ) OR ALL ( 2019-ncov AND disease ) OR ALL ( 2019 ncov AND disease ) OR ALL ( sars AND coronavirus 2 infection ) OR ALL ( sars-cov-2 AND infection ) OR ALL ( sars AND cov 2 infection ) OR ALL ( sars-cov-2 ) OR ALL ( severe AND acute AND respiratory AND syndrome AND coronavirus 2 ) OR ALL ( sars AND coronavirus 2 ) OR ALL ( covid-19 AND vaccines ) OR ALL ( covid 19 vaccine ) OR ALL ( covid-19 AND virus AND vaccine ) OR ALL ( covid 19 virus AND vaccine ) OR ALL ( sars-cov-2 AND vaccine ) OR ALL ( sars AND cov 2 vaccine ) OR ALL ( sars2 AND vaccine ) OR ALL ( coronavirus AND disease 2019 vaccine ) OR ALL ( coronavirus AND disease-19 AND vaccine ) OR ALL ( 2019-ncov AND vaccine ) OR ALL ( 2019 ncov AND vaccine ) OR ALL ( 2019 novel AND coronavirus AND vaccine ) OR ALL ( sars AND coronavirus 2 vaccine ) AND ALL ( takotsubo AND cardiomyopathy ) OR ALL ( takotsubo AND cardiomyopathy ) OR TITLE-ABS-KEY ( stress AND cardiomyopathy ) OR ALL ( tako-tsubo AND cardiomyopathy ) OR ALL ( tako AND tsubo AND cardiomyopathy ) OR ALL ( broken AND heart AND syndrome ) OR ALL ( takotsubo AND syndrome ) OR ALL ( transient AND apical AND ballooning AND syndrome ) OR ALL ( apical AND ballooning AND syndrome ) OR ALL ( left AND ventricular AND apical AND ballooning AND syndrome ) OR ALL ( tako-tsubo AND syndrome ) OR ALL ( tako AND tsubo AND syndrome ) ) AND ( LIMIT-TO ( LANGUAGE,"English" ) ) AND ( LIMIT-TO ( DOCTYPE,"ar" ) OR LIMIT-TO ( DOCTYPE,"le" ) OR LIMIT-TO ( DOCTYPE,"ed" ) OR LIMIT-TO ( DOCTYPE,"sh" ) ) | 559 |

**Table S2.** Quality assessment of the case report studies.

| Overall appraisal | 8. Does the case report provide takeaway lessons? | 7. Were adverse events (harms) or unanticipated events identified and described? | 6. Was the post-intervention clinical condition clearly described? | 5. Was the intervention(s) or treatment procedure(s) clearly described? | 4. Were diagnostic tests or assessment methods and the results clearly described? | 3. Was the current clinical condition of the patient on presentation clearly described? | 2. Was the patient’s history clearly described and presented as a timeline? | 1. Were patient’s demographic characteristics clearly described? | Study, Year |
| --- | --- | --- | --- | --- | --- | --- | --- | --- | --- |
| 6 | **✓** | **🗶** | **🗶** | **✓** | **✓** | **✓** | **✓** | **✓** | Nguyen et al., 2020 (1) |
| 8 | **✓** | **✓** | **✓** | **✓** | **✓** | **✓** | **✓** | **✓** | Panchal et al., 2020 (2) |
| 8 | **✓** | **✓** | **✓** | **✓** | **✓** | **✓** | **✓** | **✓** | Kariyanna et al., 2020 (3) |
| 7 | **✓** | **✓** | **🗶** | **✓** | **✓** | **✓** | **✓** | **✓** | Alizadehasl et al., 2021 (4) |
| 8 | **✓** | **✓** | **✓** | **✓** | **✓** | **✓** | **✓** | **✓** | Fujisaki et al., 2021 (5) |
| 8 | **✓** | **✓** | **✓** | **✓** | **✓** | **✓** | **✓** | **✓** | Torabi et al., 2020 (6) |
| 8 | **✓** | **✓** | **✓** | **✓** | **✓** | **✓** | **✓** | **✓** | Ortuno et al., 2021 (7) |
| 8 | **✓** | **✓** | **✓** | **✓** | **✓** | **✓** | **✓** | **✓** | Alshamam et al., 2021 (8) |
| 8 | **✓** | **✓** | **✓** | **✓** | **✓** | **✓** | **✓** | **✓** | Bernardi et al., 2020 (9) |
| 8 | **✓** | **✓** | **✓** | **✓** | **✓** | **✓** | **✓** | **✓** | Sattar et al., 2020 (10) |
| 8 | **✓** | **✓** | **✓** | **✓** | **✓** | **✓** | **✓** | **✓** | Tsao et al., 2020 (11) |
| 8 | **✓** | **✓** | **✓** | **✓** | **✓** | **✓** | **✓** | **✓** | Gomez et al., 2020 (12) |
| 7 | **✓** | **✓** | **✓** | **✓** | **✓** | **🗶** | **✓** | **✓** | Belli et al., 2021 (13) |
| 8 | **✓** | **✓** | **✓** | **✓** | **✓** | **✓** | **✓** | **✓** | Titi et al., 2020 (14) |
| 8 | **✓** | **✓** | **✓** | **✓** | **✓** | **✓** | **✓** | **✓** | Faqihi et al., 2020 (15) |
| 8 | **✓** | **✓** | **✓** | **✓** | **✓** | **✓** | **✓** | **✓** | Solano, López et al., 2020 (16) |
| 8 | **✓** | **✓** | **✓** | **✓** | **✓** | **✓** | **✓** | **✓** | Koh et al., 2021 (17) |
| 8 | **✓** | **✓** | **✓** | **✓** | **✓** | **✓** | **✓** | **✓** | Dave et al., 2020 (18) |
| 8 | **✓** | **✓** | **✓** | **✓** | **✓** | **✓** | **✓** | **✓** | Van Osch et al., 2020 (19) |
| 7 | **✓** | **✓** | **✓** | **🗶** | **✓** | **✓** | **✓** | **✓** | Bhattacharyya et al., 2020 (20) |
| 8 | **✓** | **✓** | **✓** | **✓** | **✓** | **✓** | **✓** | **✓** | Taza et al., 2020 (21) |
| 7 | **✓** | **✓** | **✓** | **✓** | **✓** | **✓** | **🗶** | **✓** | Bottiroli et al., 2020 (22) |
| 8 | **✓** | **✓** | **✓** | **✓** | **✓** | **✓** | **✓** | **✓** | Roca et al., 2020 (23) |
| 6 | **✓** | **✓** | **✓** | **🗶** | **✓** | **🗶** | **✓** | **✓** | Oyarzabal et al., 2020 (24) |
| 8 | **✓** | **✓** | **✓** | **✓** | **✓** | **✓** | **✓** | **✓** | Minhas et al., 2020 (25) |
| 7 | **✓** | **✓** | **✓** | **🗶** | **✓** | **✓** | **✓** | **✓** | Meyer et al., 2020 (26) |
| 8 | **✓** | **✓** | **✓** | **✓** | **✓** | **✓** | **✓** | **✓** | Habedank et al., 2020 (27) |
| 8 | **✓** | **✓** | **✓** | **✓** | **✓** | **✓** | **✓** | **✓** | Giannitsi et al., 2020 (28) |
| 6 | **✓** | **✓** | **🗶** | **✓** | **✓** | **🗶** | **✓** | **✓** | Parker et al., 2020 (29) |
| 8 | **✓** | **✓** | **✓** | **✓** | **✓** | **✓** | **✓** | **✓** | Uhe et al., 2020 (30) |
| 6 | **🗶** | **✓** | **✓** | **🗶** | **✓** | **✓** | **✓** | **✓** | Chadha et al., 2020 (31) |
| 5 | **✓** | **✓** | **🗶** | **🗶** | **✓** | **✓** | **🗶** | **✓** | Rivers et al., 2020 (32) |
| 8 | **✓** | **✓** | **✓** | **✓** | **✓** | **✓** | **✓** | **✓** | Koutroumpakis et al., 2020 (33) |
| 6 | **✓** | **✓** | **🗶** | **🗶** | **✓** | **✓** | **✓** | **✓** | Dolci et al., 2020 (34) |
| 8 | **✓** | **✓** | **✓** | **✓** | **✓** | **✓** | **✓** | **✓** | Mohammed et al., 2020 (35) |
| 5 | **✓** | **🗶** | **🗶** | **🗶** | **✓** | **✓** | **✓** | **✓** | Boscolo Berto et al., 2021 (36) |
| 8 | **✓** | **✓** | **✓** | **✓** | **✓** | **✓** | **✓** | **✓** | Fearon et al., 2021 (37) |
| 7 | **✓** | **✓** | **✓** | **🗶** | **✓** | **✓** | **✓** | **✓** | Mishra et al., 2021 (38) |
| 8 | **✓** | **✓** | **✓** | **✓** | **✓** | **✓** | **✓** | **✓** | Namburu et al., 2021 (39) |
| 6 | **✓** | **🗶** | **✓** | **🗶** | **✓** | **✓** | **✓** | **✓** | Rivera et al., 2020 (40) |
| 8 | **✓** | **✓** | **✓** | **✓** | **✓** | **✓** | **✓** | **✓** | Wildermann et al., 2022 (41) |
| 8 | **✓** | **✓** | **✓** | **✓** | **✓** | **✓** | **✓** | **✓** | Ben Ammar et al., 2021 (42) |
| 8 | **✓** | **✓** | **✓** | **✓** | **✓** | **✓** | **✓** | **✓** | Stewart et al., 2021 (43) |
| 8 | **✓** | **✓** | **✓** | **✓** | **✓** | **✓** | **✓** | **✓** | Tedeschi et al., 2022 (44) |
| 8 | **✓** | **✓** | **✓** | **✓** | **✓** | **✓** | **✓** | **✓** | Toida et al., 2022 (45) |
| 8 | **✓** | **✓** | **✓** | **✓** | **✓** | **✓** | **✓** | **✓** | Yamaura et al., 2022 (46) |
| 8 | **✓** | **✓** | **✓** | **✓** | **✓** | **✓** | **✓** | **✓** | Bapat et al., 2020 (47) |
| 8 | **✓** | **✓** | **✓** | **✓** | **✓** | **✓** | **✓** | **✓** | Chao et al., 2020 (48) |
| 8 | **✓** | **✓** | **✓** | **✓** | **✓** | **✓** | **✓** | **✓** | Dabbagh et al., 2020 (49) |
| 8 | **✓** | **✓** | **✓** | **✓** | **✓** | **✓** | **✓** | **✓** | Manzur-Sandoval et al., 2020 (50) |
| 8 | **✓** | **✓** | **✓** | **✓** | **✓** | **✓** | **✓** | **✓** | Sang et al., 2020 (51) |
| 8 | **✓** | **✓** | **✓** | **✓** | **✓** | **✓** | **✓** | **✓** | Crane et al., 2021 (52) |
| 8 | **✓** | **✓** | **✓** | **✓** | **✓** | **✓** | **✓** | **✓** | Eftekharzadeh et al., 2022 (53) |
| 7 | **✓** | **🗶** | **✓** | **✓** | **✓** | **✓** | **✓** | **✓** | Frynas-Jończyk et al., 2022 (54) |
| 8 | **✓** | **✓** | **✓** | **✓** | **✓** | **✓** | **✓** | **✓** | Fujiyoshi et al., 2022 (55) |
| 8 | **✓** | **✓** | **✓** | **✓** | **✓** | **✓** | **✓** | **✓** | Kimura et al., 2021 (56) |

Yes=✓ No=🗶 Unclear=U/C Not applicable=N/A

**Table S3.** Quality assessment of the case series.

| Overall appraisal | 10. Was statistical analysis appropriate? | 9. Was there clear reporting of the presenting site(s)/clinic(s) demographic information? | 8. Were the outcomes or follow up results of cases clearly reported? | 7. Was there clear reporting of clinical information of the participants? | 6. Was there clear reporting of the demographics of the participants in the study? | 5. Did the case series have complete inclusion of participants? | 4. Did the case series have consecutive inclusion of participants? | 3. Were valid methods used for identification of the condition for all participants d in the case series? | 2. Was the condition measured in a standard, reliable way for all participants d in the case series? | 1. Were there clear criteria for inclusion in the case series? | Study, Year |
| --- | --- | --- | --- | --- | --- | --- | --- | --- | --- | --- | --- |
| 7 | N/A | **✓** | **✓** | **✓** | **✓** | **U/C** | **U/C** | **✓** | **✓** | **✓** | Demertzis et al., 2020 (57) |
| 10 | **✓** | **✓** | **✓** | **✓** | **✓** | **✓** | **✓** | **✓** | **✓** | **✓** | Hegde et al., 2020 (58) |
| 9 | N/A | **✓** | **✓** | **✓** | **✓** | **✓** | **✓** | **✓** | **✓** | **✓** | Hoepler et al., 2021 (59) |
| 8 | N/A | **✓** | **✓** | **✓** | **✓** | **U/C** | **✓** | **✓** | **✓** | **✓** | Pasqualetto et al., 2020 (60) |
| 7 | N/A | **✓** | **✓** | **✓** | **✓** | **🗶** | **🗶** | **✓** | **✓** | **✓** | Kong et al., 2021 (61) |
| 7 | N/A | **✓** | **✓** | **✓** | **✓** | **U/C** | **U/C** | **✓** | **✓** | **✓** | Park et al., 2020 (62) |
| 7 | N/A | **✓** | **✓** | **✓** | **✓** | **U/C** | **U/C** | **✓** | **✓** | **✓** | Moady et al., 2021 (63) |
| 7 | N/A | **✓** | **✓** | **✓** | **✓** | **U/C** | **U/C** | **✓** | **✓** | **✓** | Kir et al., 2021 (64) |
| 6 | N/A | **✓** | **✓** | **✓** | **✓** | **U/C** | **U/C** | **✓** | **🗶** | **✓** | Vidula et al., 2021 (65) |
| 6 | N/A | **✓** | **✓** | **✓** | **✓** | **U/C** | **U/C** | **✓** | **✓** | **✓** | Tutor et al., 2021 (66) |

Yes=✓ No=🗶 Unclear=U/C Not applicable=N/A

**Table S4.** Quality assessment of the cohort study.

| Overall appraisal | 11. Was appropriate statistical analysis used? | 10. Were strategies to address incomplete follow up utilized? | 9. Was follow up complete, and if not, were the reasons to loss to follow up described and explored? | 8. Was the follow up time reported and sufficient to be long enough for outcomes to occur? | 7. Were the outcomes measured in a valid and reliable way? | 6. Were the groups/participants free of the outcome at the start of the study (or at the moment of exposure)? | 5. Were strategies to deal with confounding factors stated? | 4. Were confounding factors identified? | 3. Was the exposure measured in a valid and reliable way? | 2. Were the exposures measured similarly to assign people to both exposed and unexposed groups? | 1. Were the two groups similar and recruited from the same population? | Study, Year |
| --- | --- | --- | --- | --- | --- | --- | --- | --- | --- | --- | --- | --- |
| 11 | **✓** | **✓** | **✓** | **✓** | **✓** | **✓** | **✓** | **✓** | **✓** | **✓** | **✓** | Jabri et al., 2020 (67) |

Yes=✓ No=🗶 Unclear=U/C Not applicable=N/A

**References**

1. Nguyen D, Nguyen T, De Bels D, Castro Rodriguez J. A case of Takotsubo cardiomyopathy with COVID 19. European Heart Journal - Cardiovascular Imaging. 2020;21(9):1052-.

2. Panchal A, Kyvernitakis A, Biederman R. An Interesting Case of COVID-19 Induced Reversed Takotsubo Cardiomyopathy and Insight on Cardiac Biomarkers. Cureus. 2020;12(11):e11296.

3. Kariyanna PT, Chandrakumar HP, Jayarangaiah A, Khan A, Vulkanov V, Ashamalla M, et al. Apical Takotsubo Cardiomyopathy in a COVID-19 Patient Presenting with Stroke: A Case Report and Pathophysiologic Insights. Am J Med Case Rep. 2020;8(10):350-7.

4. Alizadehasl A, Soleimani A, Peighambari MM, Mostafavi A. Biventricular apical ballooning in patient with COVID-19. Journal of Echocardiography. 2021.

5. Fujisaki T, Kassim F, Kassim G, Bandyopadhyay D, Singh V, Kim B. Biventricular takotsubo syndrome with COVID-19 in an Asian male. Journal of Cardiology Cases. 2021;24(1):6-9.

6. Torabi AJ, Villegas-Galaviz J, Guglin M, Frick K, Rao R. Cardiogenic shock following cardiac tamponade and Takotsubo in COVID-19. Future Cardiology. 2020;17(4):631-5.

7. Ortuno S, Jozwiak M, Mira J-P, Nguyen LS. Case Report: Takotsubo Syndrome Associated With Novel Coronavirus Disease 2019. Frontiers in Cardiovascular Medicine. 2021;8.

8. Alshamam MS, Nso N, Idrees Z, Nassar M, Munira MS. Coronavirus Disease 2019 (COVID-19)-Induced Takotsubo Cardiomyopathy Prognosis in Geriatric Setting. Cureus. 2021;13(7):e16211.

9. Bernardi N, Calvi E, Cimino G, Pascariello G, Nardi M, Cani D, et al. COVID-19 Pneumonia, Takotsubo Syndrome, and Left Ventricle Thrombi. JACC Case Rep. 2020;2(9):1359-64.

10. Sattar Y, Connerney M, Ullah W, Philippou A, Slack D, McCarthy B, et al. COVID-19 Presenting as Takotsubo Cardiomyopathy Complicated with Atrial Fibrillation. Int J Cardiol Heart Vasc. 2020;29:100580.

11. Tsao CW, Strom JB, Chang JD, Manning WJ. COVID-19-Associated Stress (Takotsubo) Cardiomyopathy. Circ Cardiovasc Imaging. 2020;13(7):e011222.

12. Gomez JMD, Nair G, Nanavaty P, Rao A, Marinescu K, Suboc T. COVID-19-associated takotsubo cardiomyopathy. BMJ Case Rep. 2020;13(12).

13. Belli O, Ardissino M, Bottiroli M, Soriano F, Blanda C, Oreglia J, et al. Emergency cardiac imaging for coronavirus disease 2019 (COVID-19) in practice: a case of takotsubo stress cardiomyopathy. Cardiovascular Ultrasound. 2021;19(1).

14. Titi L, Magnanimi E, Mancone M, Infusino F, Coppola G, Del Nonno F, et al. Fatal Takotsubo syndrome in critical COVID-19 related pneumonia. Cardiovasc Pathol. 2021;51:107314.

15. Faqihi F, Alharthy A, Alshaya R, Papanikolaou J, Kutsogiannis DJ, Brindley PG, et al. Reverse takotsubo cardiomyopathy in fulminant COVID-19 associated with cytokine release syndrome and resolution following therapeutic plasma exchange: a case-report. BMC Cardiovasc Disord. 2020;20(1):389.

16. Solano-López J, Sánchez-Recalde A, Zamorano JL. SARS-CoV-2, a novel virus with an unusual cardiac feature: inverted takotsubo syndrome. Eur Heart J. 2020;41(32):3106.

17. Koh MCY, Li TYW, Ong JSY, Somani J, Ambhore AA. Stress Cardiomyopathy with Transient Biventricular Dysfunction Following Recent COVID-19 Infection. Acta Cardiol Sin. 2021;37(2):204-7.

18. Dave S, Thibodeau JT, Styrvoky K, Bhatt SH. Takotsubo Cardiomyopathy in a Coronavirus Disease-2019-Positive Patient: A Case Report. A A Pract. 2020;14(11):e01304.

19. van Osch D, Asselbergs FW, Teske AJ. Takotsubo cardiomyopathy in COVID-19: a case report. Haemodynamic and therapeutic considerations. Eur Heart J Case Rep. 2020;4(Fi1):1-6.

20. Bhattacharyya PJ, Attri PK, Farooqui W. Takotsubo cardiomyopathy in early term pregnancy: a rare cardiac complication of SARS-CoV-2 infection. BMJ Case Rep. 2020;13(9).

21. Taza F, Zulty M, Kanwal A, Grove D. Takotsubo cardiomyopathy triggered by SARS-CoV-2 infection in a critically ill patient. BMJ Case Rep. 2020;13(6).

22. Bottiroli M, De Caria D, Belli O, Calini A, Andreoni P, Siragusa A, et al. Takotsubo syndrome as a complication in a critically ill COVID-19 patient. ESC Heart Fail. 2020;7(6):4297-300.

23. Roca E, Lombardi C, Campana M, Vivaldi O, Bigni B, Bertozzi B, et al. Takotsubo Syndrome Associated with COVID-19. Eur J Case Rep Intern Med. 2020;7(5):001665.

24. Oyarzabal L, Gómez-Hospital JA, Comin-Colet J. Tako-tsubo syndrome associated with COVID-19. Rev Esp Cardiol (Engl Ed). 2020;73(10):846.

25. Minhas AS, Scheel P, Garibaldi B, Liu G, Horton M, Jennings M, et al. Takotsubo Syndrome in the Setting of COVID-19. JACC Case Rep. 2020;2(9):1321-5.

26. Meyer P, Degrauwe S, Van Delden C, Ghadri JR, Templin C. Typical takotsubo syndrome triggered by SARS-CoV-2 infection. Eur Heart J. 2020;41(19):1860.

27. Habedank D, Thieme R, Bublak A, Heinemann F, Spencker S, Atmowihardjo I. Ventricular fibrillation and Takotsubo cardiomyopathy triggered by media panic on COVID-19: A case report. Clin Case Rep. 2021;9(1):72-6.

28. Giannitsi S, Tsinivizov P, Poulimenos LE, Kallistratos MS, Varvarousis D, Manolis AJ, et al. [Case Report] Stress induced (Takotsubo) cardiomyopathy triggered by the COVID-19 pandemic. Exp Ther Med. 2020;20(3):2812-4.

29. Parker J, Niranjan S, Sriram KB. A case of broken heart syndrome via the telephone: socially distant outpatient clinics in the COVID-19 pandemic. Intern Med J. 2020;50(11):1429-31.

30. Uhe T, Hagendorff A, Wachter R, Laufs U. Collateral damage: Fear from SARS-CoV2-infection causing Takotsubo cardiomyopathy. Clin Res Cardiol. 2020;109(12):1588-94.

31. Chadha S. 'COVID-19 pandemic' anxiety-induced Takotsubo cardiomyopathy. Qjm. 2020;113(7):488-90.

32. Rivers J, Ihle JF. COVID-19 social isolation-induced takotsubo cardiomyopathy. Med J Aust. 2020;213(7):336-.e1.

33. Koutroumpakis E, Taylor T, Damaraju S, Badruddin Mawji S. "Covidsubo": Stress-Induced Cardiomyopathy by Novel Coronavirus Disease 2019. Cardiology. 2020;145(12):779-83.

34. Dolci G, Prevedello F, Lobascio I, Mugnai G, Dalla Valle C, Bilato C. Mid-ventricular Takotsubo syndrome 'lockdown'-related during coronavirus disease 2019 outbreak: a case report. J Cardiovasc Med (Hagerstown). 2021;22(5):414-6.

35. Mohammed M, Zakhour S, Devgun J, Lee J, Keimig T, Wang DD. Takotsubo Cardiomyopathy in a Healthcare Worker During the COVID-19 Pandemic: Caused by the Virus or the Demands of the Many Being Placed on the Few? Eur J Case Rep Intern Med. 2020;7(12):002088.

36. Boscolo Berto M, Spano G, Wagner B, Bernhard B, Häner J, Huber AT, et al. Takotsubo Cardiomyopathy After mRNA COVID-19 Vaccination. Heart Lung Circ. 2021;30(12):e119-e20.

37. Fearon C, Parwani P, Gow-Lee B, Abramov D. Takotsubo syndrome after receiving the COVID-19 vaccine. J Cardiol Cases. 2021;24(5):223-6.

38. Mishra AK, Dai Q, Sahu KK, ElMeligy A. Atypical Takotsubo Cardiomyopathy in COVID-19. Am J Med Sci. 2021;362(5):e41-e2.

39. Namburu L, Bhogal SS, Ramu VK. COVID-19-Induced Takotsubo Cardiomyopathy With Concomitant Pulmonary Embolism. Cureus. 2021;13(10):e18693.

40. Rivera K, Fernández-Rodríguez D, Zielonka M, Casanova-Sandoval J. Diagnosis of Takotsubo syndrome in the COVID-19 era. Rev Port Cardiol. 2021;40(11):899-901.

41. Wildemann B, Jarius S, Lehmann LH, André F, Frey N, Schnitzler P, et al. COVID-19-related severe MS exacerbation with life-threatening Takotsubo cardiomyopathy in a previously stable patient and interference of MS therapy with long-term immunity against SARS-CoV-2. J Neurol. 2022;269(3):1138-41.

42. Ben Ammar H, Bouguira E, Brahmi L, Hamdi G, Bouallagui A, Khelifa E, et al. Simultaneous occurrence of a Takotsubo syndrome and paranoia delirium, related to Covid-19 pandemic: A case report. Clin Case Rep. 2021;9(11):e05026.

43. Stewart C, Gamble DT, Dawson D. Novel case of takotsubo cardiomyopathy following COVID-19 vaccination. BMJ Case Rep. 2022;15(1).

44. Tedeschi A, Camilli M, Ianni U, Tavecchia G, Palazzini M, Cartella I, et al. Takotsubo syndrome after BNT162b2 mRNA Covid-19 vaccine: Emotional or causative relationship with vaccination? Int J Cardiol Heart Vasc. 2022;40:101002.

45. Toida R, Uezono S, Komatsu H, Toida T, Imamura A, Fujimoto S, et al. Takotsubo cardiomyopathy after vaccination for coronavirus disease 2019 in a patient on maintenance hemodialysis. CEN Case Rep. 2022;11(2):220-4.

46. Yamaura H, Ishikawa H, Otsuka K, Kasayuki N. Reverse Takotsubo Cardiomyopathy as a Cause of Acute Chest Pain in a Young Woman Following COVID-19 Vaccination. Circ Cardiovasc Imaging. 2022;15(1):e013661.

47. Bapat A, Maan A, Heist EK. Stress-Induced Cardiomyopathy Secondary to COVID-19. Case Rep Cardiol. 2020;2020:8842150.

48. Chao CJ, DeValeria PA, Sen A, Lee H, Pedrotty DM, Patel B, et al. Reversible cardiac dysfunction in severe COVID-19 infection, mechanisms and case report. Echocardiography. 2020;37(9):1465-9.

49. Dabbagh MF, Aurora L, D'Souza P, Weinmann AJ, Bhargava P, Basir MB. Cardiac Tamponade Secondary to COVID-19. JACC Case Rep. 2020;2(9):1326-30.

50. Manzur-Sandoval D, Carmona-Levario P, García-Cruz E. Giant inverted T waves in a patient with COVID-19 infection. Ann Emerg Med. 2021;77(2):264-7.

51. Sang CJ, 3rd, Heindl B, Von Mering G, Brott B, Kopf RS, Benson PV, et al. Stress-Induced Cardiomyopathy Precipitated by COVID-19 and Influenza A Coinfection. JACC Case Rep. 2020;2(9):1356-8.

52. Crane P, Wong C, Mehta N, Barlis P. Takotsubo (stress) cardiomyopathy after ChAdOx1 nCoV-19 vaccination. BMJ Case Rep. 2021;14(10).

53. Eftekharzadeh P, Patel A, Sokolova E, Rodas A, Ahmed S. Takotsubo Cardiomyopathy: A COVID-19 Complication. Cureus. 2022;14(3):e22803.

54. Frynas-Jończyk K, Ćwiek-Rębowska E, Filipiak-Strzecka D, Szymczyk E, Kasprzak JD. COVID-tsubo: takotsubo syndrome in patient hospitalized due to the SARS-CoV-2 infection. Pol Arch Intern Med. 2022.

55. Fujiyoshi K, Ako J, Ishida K, Ishida M, Minami Y, Inomata T. Tako-tsubo-like left ventricular dysfunction in a patient with COVID-19 demonstrated by non-invasive multi-modality imaging. J Nucl Cardiol. 2022;29(2):863-5.

56. Kimura M, Hashiguchi S, Tanaka K, Hagiwara M, Takahashi K, Miyaji Y, et al. Case Report: Takotsubo Cardiomyopathy in Bickerstaff Brainstem Encephalitis Triggered by COVID-19. Front Neurol. 2021;12:822247.

57. Demertzis ZD, Dagher C, Malette KM, Fadel RA, Bradley PB, Brar I, et al. Cardiac sequelae of novel coronavirus disease 2019 (COVID-19): a clinical case series. European Heart Journal - Case Reports. 2020;4(FI1):1-6.

58. Hegde S, Khan R, Zordok M, Maysky M. Characteristics and outcome of patients with COVID-19 complicated by Takotsubo cardiomyopathy: case series with literature review. Open Heart. 2020;7(2):e001360.

59. Hoepler W, Traugott MT, Christ G, Kitzberger R, Pawelka E, Karolyi M, et al. Clinical and Angiographic Features in Three COVID-19 Patients with Takotsubo Cardiomyopathy. Case Report. SN Comprehensive Clinical Medicine. 2021;3(1):263-8.

60. Pasqualetto MC, Secco E, Nizzetto M, Scevola M, Altafini L, Cester A, et al. Stress Cardiomyopathy in COVID-19 Disease. Eur J Case Rep Intern Med. 2020;7(6):001718.

61. Kong N, Singh N, Mazzone S, Burkhart R, Anchan R, Blair J. Takotsubo Syndrome Presenting as Cardiogenic Shock in Patients With COVID-19: A Case Series and Review of Current Literature. Cardiovasc Revasc Med. 2021;28s:50-3.

62. Park JH, Moon JY, Sohn KM, Kim YS. Two Fatal Cases of Stress-induced Cardiomyopathy in COVID-19 Patients. J Cardiovasc Imaging. 2020;28(4):300-3.

63. Moady G, Atar S. Quarantine-induced Stress Cardiomyopathy (Takotsubo syndrome) during the COVID-19 pandemic. Isr Med Assoc J. 2021;23(3):149-52.

64. Kir D, Beer N, De Marchena EJ. Takotsubo cardiomyopathy caused by emotional stressors in the coronavirus disease 2019 (COVID-19) pandemic era. J Card Surg. 2021;36(2):764-9.

65. Vidula MK, Ambrose M, Glassberg H, Chokshi N, Chen T, Ferrari VA, et al. Myocarditis and Other Cardiovascular Complications of the mRNA-Based COVID-19 Vaccines. Cureus. 2021;13(6):e15576.

66. Tutor A, Unis G, Ruiz B, Bolaji OA, Bob-Manuel T. Spectrum of Suspected Cardiomyopathy Due to COVID-19: A Case Series. Curr Probl Cardiol. 2021;46(10):100926.

67. Jabri A, Kalra A, Kumar A, Alameh A, Adroja S, Bashir H, et al. Incidence of Stress Cardiomyopathy During the Coronavirus Disease 2019 Pandemic. JAMA Netw Open. 2020;3(7):e2014780.
